# Knocking out alpha-synuclein in melanoma cells downregulates L1CAM and decreases motility

**DOI:** 10.1038/s41598-023-36451-3

**Published:** 2023-06-07

**Authors:** Nithya Gajendran, Santhanasabapathy Rajasekaran, Stephan N. Witt

**Affiliations:** 1 Department of Biochemistry and Molecular Biology, Louisiana State University Health Sciences Center, Shreveport, USA; 2 Feist-Weiller Cancer Center, Louisiana State University Health Shreveport, Shreveport, USA

**Keywords:** Mechanisms of disease, Membrane trafficking, Skin cancer, Cancer, Cell biology

## Abstract

The Parkinson’s disease (PD) associated protein, alpha-synuclein (α-syn/*SNCA*), is highly expressed in aggressive melanomas. The goal of this study was to reveal possible mechanism(s) of α-syn involvement in melanoma pathogenesis. Herein, we asked whether α-syn modulates the expression of the pro-oncogenic adhesion molecules L1CAM and N-cadherin. We used two human melanoma cell lines (SK-MEL-28, SK-MEL-29), *SNCA*-knockout (KO) clones, and two human SH-SY5Y neuroblastoma cell lines. In the melanoma lines, loss of α-syn expression resulted in significant decreases in the expression of L1CAM and N-cadherin and concomitant significant decreases in motility. On average, there was a 75% reduction in motility in the four *SNCA*-KOs tested compared to control cells. Strikingly, comparing neuroblastoma SH-SY5Y cells that have no detectable α-syn to SH-SY5Y cells that stably express α-syn (SH/+αS), we found that expressing α-syn increased L1CAM and single-cell motility by 54% and 597%, respectively. The reduction in L1CAM level in *SNCA*-KO clones was not due to a transcriptional effect, rather we found that L1CAM is more efficiently degraded in the lysosome in *SNCA*-KO clones than in control cells. We propose that α-syn is pro-survival to melanoma (and possibly neuroblastoma) because it promotes the intracellular trafficking of L1CAM to the plasma membrane.

## Introduction

Melanoma is an aggressive skin cancer that arises from pigment-producing cells called melanocytes. Epidemiological studies have reported the co-occurrence of melanoma and Parkinson’s disease (PD)^1,2^. Such a co-occurrence is unusual in that these two diseases are so different in that melanoma is characterized by uncontrolled cell proliferation whereas PD by neuronal cell death. Melanoma patients have a 1.5- to 1.85-fold higher risk of developing PD compared to age- and sex-matched controls^3,4^, and reciprocally, PD patients have a 1.4- to 20-fold higher risk of developing invasive melanoma than a control group^5–7^. The mechanism of this co-occurrence is unknown and could involve multiple genes^2^, one of which is *SNCA*, and even environmental factors. Here we focus on the role of *SNCA* in melanoma.

*SNCA* codes for the protein alpha-synuclein (α-syn)^8–10^, which is expressed in neurons^11–13^ and a variety of other tissues^14–16^, including melanomas^17^, where it is highly expressed. α-Syn is a small intrinsically unfolded protein that in neurons localizes to nerve terminals where synaptic vesicles are docked^18^; it accelerates the kinetics of individual exocytotic events^19^. Whether α-syn promotes exocytosis in other cell types is not known. Many other activities have been ascribed to this unique protein because it, in its multitude of conformations, binds to so many biomolecules, i.e., negatively charged phospholipids^20,21^, proteins^22–24^, and DNA^25^. Thus, while α-syn functions in the endolysosomal system to promote endocytosis and exocytosis^19,26^, and it may have other functions. The intrinsically unfolded nature of α-syn makes it highly prone to aggregate into oligomeric and prion-like, amyloidogenic conformations^27^, and some of these aggregates trigger neurodegeneration in PD^28^. Paradoxically, prion-like aggregates of α-syn have been suggested to promote autophagy in melanoma cells^29^, which is pro-survival. Whether α-syn has other pro-survival functions in melanoma is the subject of this study.

Two studies assessed the effect of knocking out *SNCA* on cellular homeostasis. First, knocking down *SNCA* in mouse retinal epithelial cells in vitro significantly decreased the level of the transferrin receptor (TfR1) and its mRNA transcript, and overexpressing α-syn increased the levels of the TfR1 protein and its mRNA transcript relative to control cells^30,31^. The loss of α-syn expression in retinal epithelial cells caused TfR1 molecules to accumulate in Golgi vesicles, suggesting that α-syn is required for the pathway that transits TfR1 from the *trans*-Golgi to the plasma membrane^30^. Second, *SNCA* was knocked out in the human cutaneous melanoma cell line, SK-MEL-28, and the *SNCA*-KO clones were assessed in vitro and in a mouse xenograft model^31^. The loss of α-syn expression in these melanoma cells decreased the levels of TfR1 and the iron exporter ferroportin and significantly suppressed the growth of the *SNCA-*KO tumors engrafted in nude mice. The reduction in the level of TfR1 in the *SNCA*-KO clones was a consequence of its enhanced lysosomal degradation. The results from these two studies are consistent with α-syn promoting the vesicular trafficking of TfR1.

In this study, we sought to determine whether the expression level of α-syn affects the expression level of adhesions proteins. Specifically, given that TfR1 and L1CAM, which is an adhesion protein expressed in neurons and melanomas, co-localize upon endocytosis in 3T3 cells^32^, we asked whether α-syn modulates the expression of L1CAM, like it does TfR1. To this end, we measured the levels of L1CAM in melanoma and neuroblastoma cells with or without the expression of α-syn. In addition, we also assessed the levels of E- and N-cadherin, and vimentin, which are three proteins involved in the epithelial-to-mesenchymal transition (EMT)^33,34^. The EMT is a highly regulated transition where epithelial cells shed their epithelial markers and morphology and convert to a mesenchymal phenotype. However, because melanocytes (and melanomas) are not epithelial cells, it is a misnomer to say that melanocytes undergo an EMT. We show that knocking out α-syn in melanoma cell lines and low expression of α-syn in a neuroblastoma cell line cause significant decreases in L1CAM compared to control cells that express α-syn. Our interpretation of these findings is that α-syn is a pro-survival factor in melanoma because it acts post-translationally to maintain a high levels of L1CAM and in turn high levels of motility.

## Results

The cell lines used in this study are given in Table 1. Each melanoma cell line harbors the BRAF V600E mutation^35^, which is the most common mutation in cutaneous melanoma (Table 1). This mutation causes constitutive activation of the RAS-RAF-MEK-ERK signaling pathway^36^, which leads to proliferation. SH-SY5Y cells, which are widely used to study PD^37^, are derived from a neuroblastoma.

**Table 1:** Cell lines used in this study.

Cell line	Comments	α-syn (Y,N)	Mutations	Ref
SK-MEL-28	This cell line was isolated from skin tissue from a 51-year-old, male with malignant melanoma	Y	BRAF V600E	^17^
*SNCA*-KO clones KO8, K09	*SNCA* knocked out in SK-MEL-28 cells using CRISPR/Cas9	N	BRAF V600E	^31^
*SNCA*-KI clones KI8, KI9	*SNCA* cDNA was re-introduced into the KO clones using lenti virus	Y	BRAF V600E	^31^
SK-MEL-29	This cell line was isolated from a recurrent melanoma at the apex of the left axilla of a 19-year-old male	Y	BRAF V600E	
*SNCA*-KO clones KO1, KO2	*SNCA* knocked out in SK-MEL-29 cells using CRISPR/Cas9	N	BRAF V600E	This study
SH-SY5Y	Cell line derived from a neuroblastoma from a 4-year-old child	N	Unknown	^61^
SH/aS	SH-SY5Y stably expressing α-syn	Y	Unknown	^61^

α-Syn expression was tested by Western blotting and qPCR. BRAF mutation status is also given.

### SK-MEL-28 *SNCA*-KO cells have decreased levels of EMT-like markers and decreased invasion, migration, and motility

**Figure 1:**
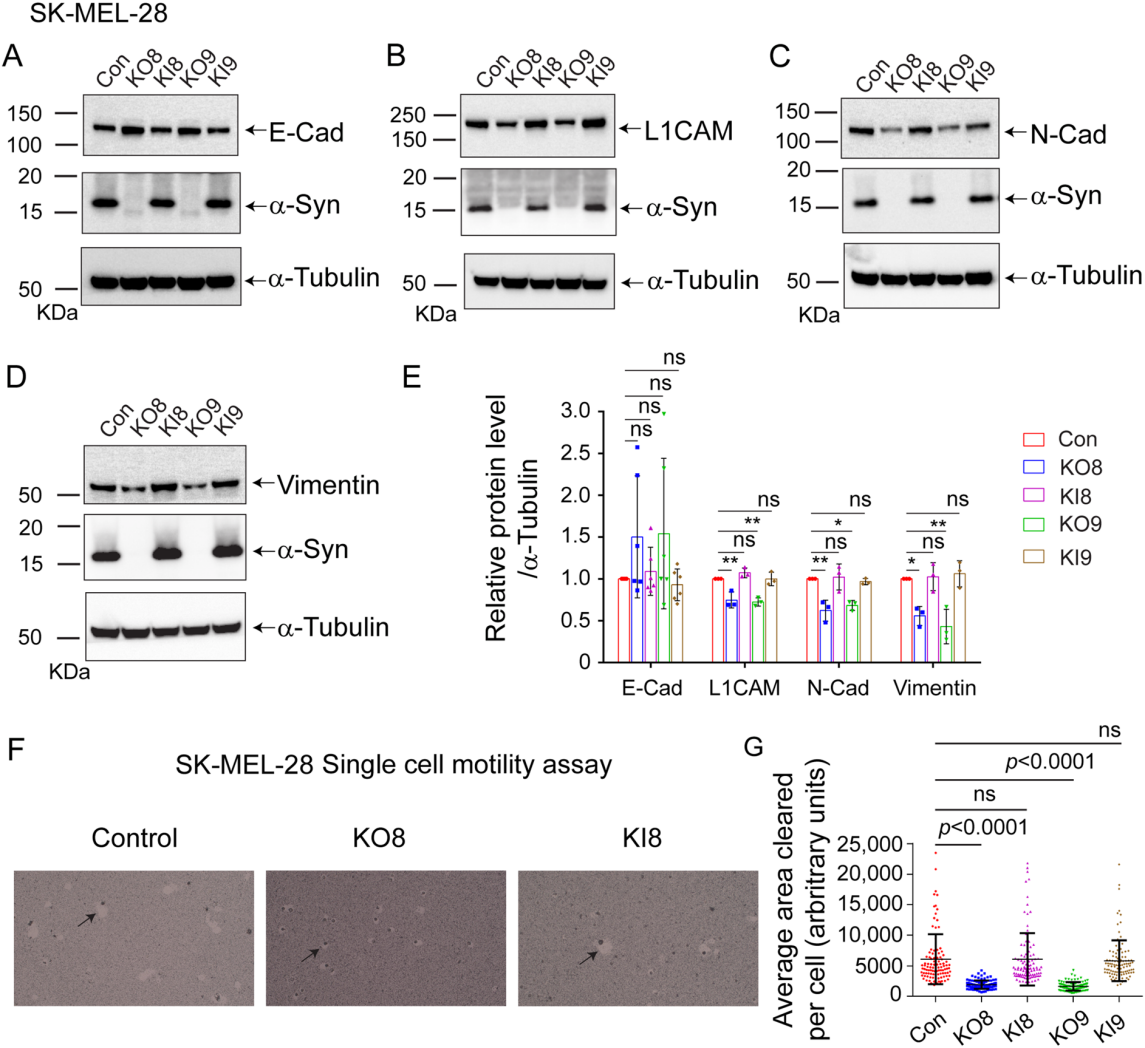
Loss of α-syn decreases EMT-like markers and motility in SK-MEL-28 cells. **(A–D**) Representative western blots of E-Cad, L1CAM, N-Cad, vimentin, α-syn, and α-tubulin in lysates of the control, KO, and KI cells cultured in vitro. (**E**) Quantitative analysis of relative protein levels. E-Cad, L1CAM, N-Cad, and vimentin band intensities are normalized to α-tubulin. All the experiments were repeated with at least three biological replicates (n = 3). (**F**) Representative light microscope images of phagokinetic tracks created by control, KO8, and KI8 cells on colloidal gold-coated wells. Black arrows mark individual phagokinetic tracks in respective cell lines. Images were acquired by using a ×10 objective, and the cleared phagokinetic area per cell was measured using ImageJ software. (**G**) Bar graphs show quantification of the cleared areas from three independent experiments. At least 33 tracks per experimental condition were randomly chosen for quantification. 100 tracks for n = 3 per experimental group were used for statistical analysis. (**E**,**G**) Values are mean ± s.d. **, *p* = 0.0015–0.0027; *, *p* = 0.0073–0.0134 determined using a one-way ANOVA, Dunnett post hoc test.

We examined SK-MEL-28 control cells and their derivatives (Table 1). The levels of E-cadherin, L1CAM, N-cadherin, vimentin, α-syn, and α-tubulin were probed in cell extracts using Western blotting (Fig. 1A–D; Supplementary Figs. S1 and S2). The normalized band intensities from a densitometric analysis of the blots are shown in Fig. 1E. Compared to the SK-MEL-28 control cells, in two KO clones the L1CAM, N-cadherin, and vimentin were downregulated by 26% (*P* = 0.002), 35% (*P* = 0.005), and 52% (*P* = 0.008), respectively. In contrast, the level of E-cadherin was unaffected (Fig. 1E). Clones KI8 and KI9 exhibited levels of these three proteins like the control cells.

Given that the *SNCA*-KO clones express lower levels of L1CAM, N-cadherin, and vimentin, which are linked to invasion and migration, we asked whether the KO clones are less motile than control cells. To this end, we used a colloidal gold single-cell motility assay^38^ to monitor the movement of single cells. As a cell moves across the surface of a slide covered with colloidal gold, the cell leaves a track where there is less gold on the surface. Representative images of the movement of control, KO8, and KI8 cells are shown in Fig. 1F, while that of control, KO9, and KI9 are shown in Supplementary Fig. S3. The control cells created much larger tracks than the KO8 clones, and the KI8 clone pattern of tracks was like that of the control cells. ImageJ was used to measure the area of the tracks, and the results are plotted in Fig. 1G. Loss of α-syn expression significantly decreased single-cell motility of KO8 and KO9 by 69% (*P* < 0.0001) and 73% (*P* < 0.0001), respectively, and re-expression of α-syn restored motility to the level of the control cells.

**Figure 2:**
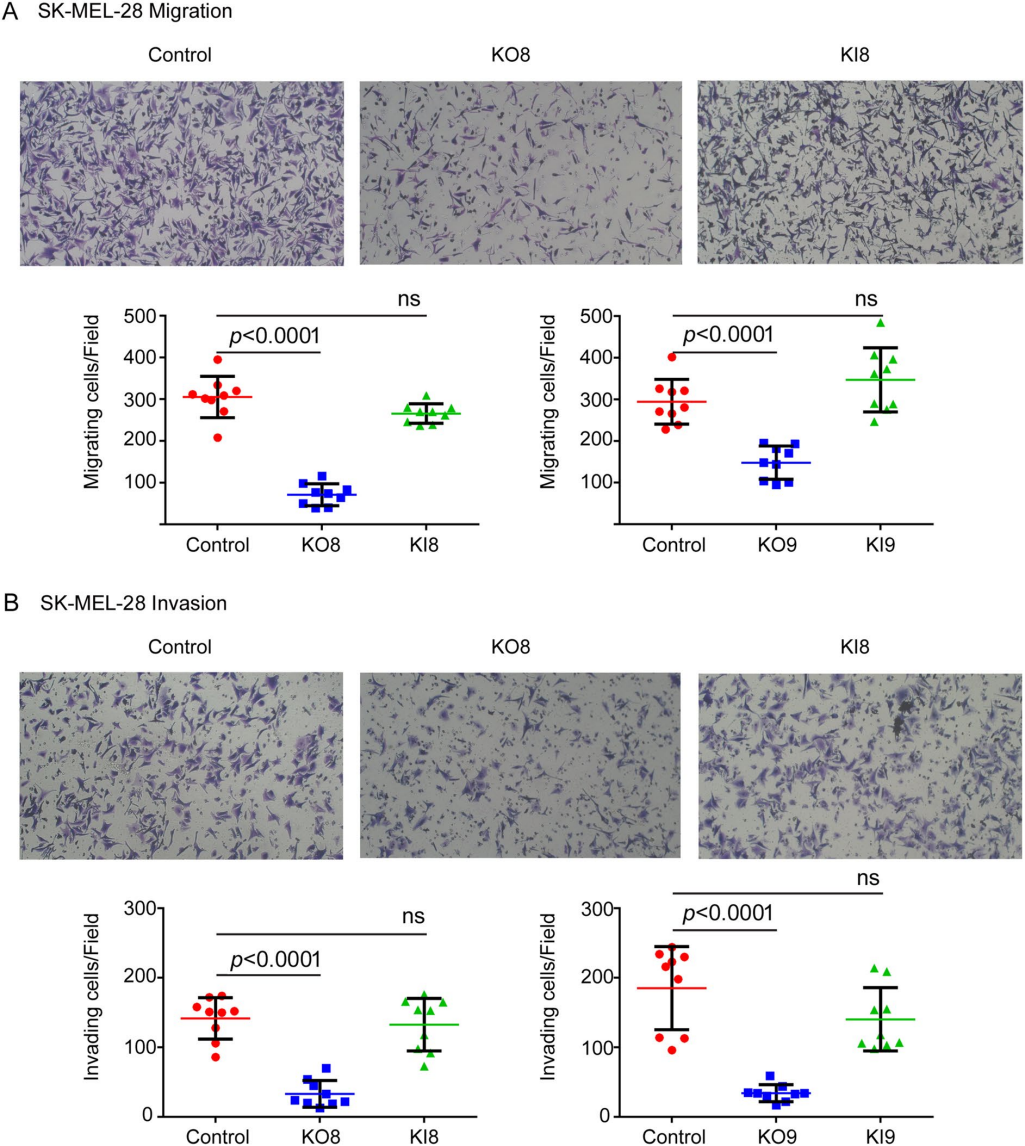
*SNCA-KO* reduces migration and invasion in SK-MEL-28 cells as assessed by transwell chamber assay. In the migration assay, 1 × 10^5^ cells in serum-free media were seeded in the upper chamber of the transwell (8 μm pore) apparatus and allowed to migrate through the membrane into the bottom chamber. In the invasion assay, 50 µl matrigel (0.2 mg/ml) was added to form a thin gel layer before the assay, and then 4 × 10^5^ cells in serum-free media were seeded in the upper chamber and allowed to invade through the membrane into the bottom chamber. In both experiments, DMEM complete media with 50% FBS in the lower chamber served as chemoattractant. Cells that passed through the membrane were fixed on the membrane with paraformaldehyde and stained with crystal violet. **(A**) Representative images of migrated control, KO8, and KI8 cells (after 24 h) per 10× field. A total of three microscopic fields were randomly selected from each inner membrane and cells were counted and represented in graph (n = 3 independent experiments). (**B**) Representative images of invaded control, KO8, and KI8 cells (after 24 h) per 10× field. A total of three microscopic fields were randomly selected from each inner membrane, and cells were counted and represented in graphs (n = 3 independent experiments). Values are mean ± s.d. *P*-values determined by a one-way ANOVA, Dunnett post hoc test.

The invasion and migration potential of SK-MEL-28 control, *SNCA*-KO, and *SNCA*-KI cells were determined by transwell assays using Boyden chambers^39^. The invasion assay measures the ability of cells in serum-free media in the top chamber to invade the matrigel and move into the lower chamber with complete media containing FBS. The migration assay uses the same setup but without matrigel. After 24 h, cells in the lower chamber were fixed, stained with crystal violet, imaged by light microscopy, and counted. Representative images from the migration assay that compared control, KO8, and KI8 are shown in Fig. 2A; images for control, KO9, and KI9 are shown in Supplementary Fig. S3. The number of migrated cells per field showed a significant 77% (*P* < 0.0001) decrease for KO8 cells compared to the control cells, and KI8 exhibited the same level of migrated cells per field as the control cells (Fig. 2A, left-hand plot). Similar results were found for the KO9 and KI9 clones (Fig. 2A, right-hand plot). Representative images from the invasion assay that compared control, KO8, and KI8 are shown in Fig. 2B; images for control, KO9, and KI9 are shown in Supplementary Fig. S3. The number of invaded cells per field showed a significant 77% (*P* < 0.0001) decrease for KO8 cells compared to control cells, and KI8 exhibited the same level of migrated cells as the control cells (Fig. 2B, left-hand plot). Similar results were obtained for the KO9 and KI9 clones (Fig. 2B, right-hand plot).

Next, we asked why the level of L1CAM is significantly lower in *SNCA*-KO cells relative to control cells. We focused on L1CAM because (i) L1CAM and α-syn has been implicated in synaptic plasticity^40^. (ii) L1CAM is recognized as a tumor antigen involved in motility^41–43^. (iii) L1CAM is endocytosed with TfR1 in 3T3 fibroblast cells^32^. (iv) TfR1 molecules are more efficiently degraded in the lysosome in *SNCA*-KO cells compared to control cells^31^. We hypothesized that α-syn promotes the efficient trafficking of endocytic vesicles containing L1CAM to and from the plasma membrane; consequently, in the absence of α-syn a large proportion of endocytic vesicles containing L1CAM molecules fail to reach the plasma membrane and instead are shunted to the lysosome for degradation. At least four pathways lead to the lysosome^44,45^: (i) endosome to lysosome pathway, (ii) phagocytic pathway, (iii) autophagy to lysosome pathway (macroautophagy), and (iv) chaperone-mediated autophagy. Given the design of the following experiments, we posit that we probed the endosome to lysosome pathway, although we also monitored a marker of autophagy.

**Figure 3:**
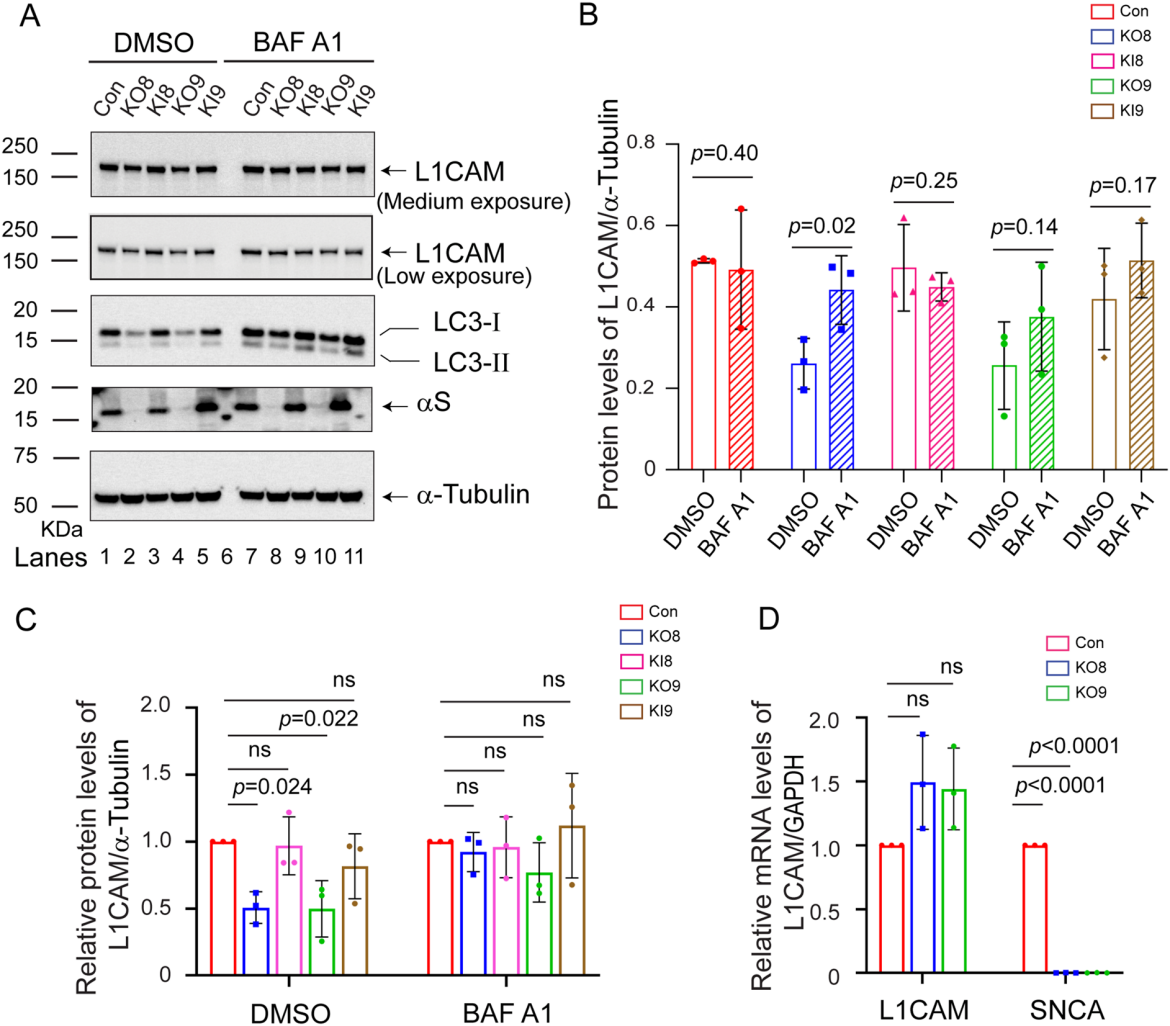
Loss of α-syn promotes the lysosomal degradation of L1CAM. (**A**) Western blot analysis of levels of L1CAM, LC3-I and -II, α-syn, and α -tubulin in lysates of control, KO and KI cells, which had been treated for 5 h with DMSO or baf. Indicated cells were treated with 50 nM baf for 5 h and the lysates were probed for the indicated proteins. Band intensities were quantified by densitometry. This experiment was conducted on n = 3 biological replicates. (**B**) Plot of L1CAM level in lysates of untreated (DMSO) versus treated (baf) cells. In this plot, L1CAM (L1) was normalized to α-tubulin (tub), according to (I_L1_/I_tub_), where I_L1_ and I_tub_ are the average intensities of the respective bands. *P*-values were determined by a one-sided Student’s t test. (**C**) Plot of L1CAM level compared by treatment group. In this plot, L1CAM was normalized to α-tubulin, according to (I_L1_/I_tub_)_sample_ (I_tub_/I_L1_)_control_. *P*-values were determined by a one-way ANOVA with Dunnett post hoc test. (**D**) Quantitative RT-PCR analysis of L1CAM. Relative mRNA levels in fold-change of L1CAM and *SNCA* normalized to housekeeping gene GAPDH. *P*-values were determined by a one-way ANOVA, Dunnett post hoc (n = 3). Values in plots (**B–D**) are mean ± s.d.

We tested for degradation of L1CAM molecules in the lysosome with the inhibitor bafilomycin A1 (baf)^46^. Baf prevents the acidification of the lysosome, hence lysosomal proteases, which require low pH for activity, are inactive. Vesicles can still merge with lysosomes in baf-treated cells, but their cargo proteins cannot be degraded. If in the absence of α-syn expression endocytic vesicles containing L1CAM molecules are shunted to the lysosome for degradation, then baf should block such degradation; thus, the level of L1CAM in baf-treated *SNCA*-KO cells should be the same as in control cells. Cells were treated for 5 h with dimethyl sulfoxide (DMSO) or baf. The cell lysates were probed for L1CAM, LC3-I and -II, α-syn, and α-tubulin by Western blotting (Fig. 3A; Supplementary Figs. S4 and S5). LC3-II, which is a lipidated form of LC3, builds up in autophagosomes if autophagy is inhibited. Band intensities were quantified by densitometry, and the resulting data were normalized and analyzed in two ways.

First, we compared the level of L1CAM in untreated versus treated cells using a one-sided t-test. A 5-h baf treatment failed to increase L1CAM in the control and the two KI clones (Fig. 3B). In contrast, baf treatment increased L1CAM by 70% (*P* = 0.02) and a 47% (*P* = 0.14) in KO8 and KO9 clones, respectively. These results show that over a 5 h period a significant number of L1CAM molecules were degraded in the lysosome of the KO8 clone but not in control cells, KO9, and KI clones.

Second, we compared the levels of L1CAM in the untreated group and separately in the baf-treated group. In this analysis, the L1CAM levels in the KO and KI clones were normalized to control cells. In the DMSO group, the expression of L1CAM in each of the *SNCA*-KO clones was decreased on average 50% (*P* = 0.022–0.024) relative to control cells (Fig. 3A, lanes 2 and 4 vs 1; Fig. 3C). In contrast, in the baf group, L1CAM showed no significant decrease in the KO clones relative to control cells (Fig. 3A, lanes 8 and 10 vs 7; Fig. 3C).

Additional control experiments were as follows. Although L1CAM is a membrane protein, and membrane proteins are degraded in the lysosome, we verified that L1CAM is not subject to proteasomal degradation using the proteasome inhibitor MG132 (Supplementary Fig. S6). We also conducted quantitative PCR (qPCR) to check the level of L1CAM mRNA in control and KO clones. No evidence was found for a decrease in L1CAM mRNA in KO8 and KO9 cells compared to the control cells. Instead, a modest increase in this transcript in the KO clones was detected (Fig. 3D). The combined results are consistent with a decrease in the level of L1CAM in the *SNCA*-KO cells due to the degradation of the protein in the lysosome.

### SK-MEL-29 *SNCA*-KO cells have decreased levels of EMT-like markers and decreased motility

**Figure 4:**
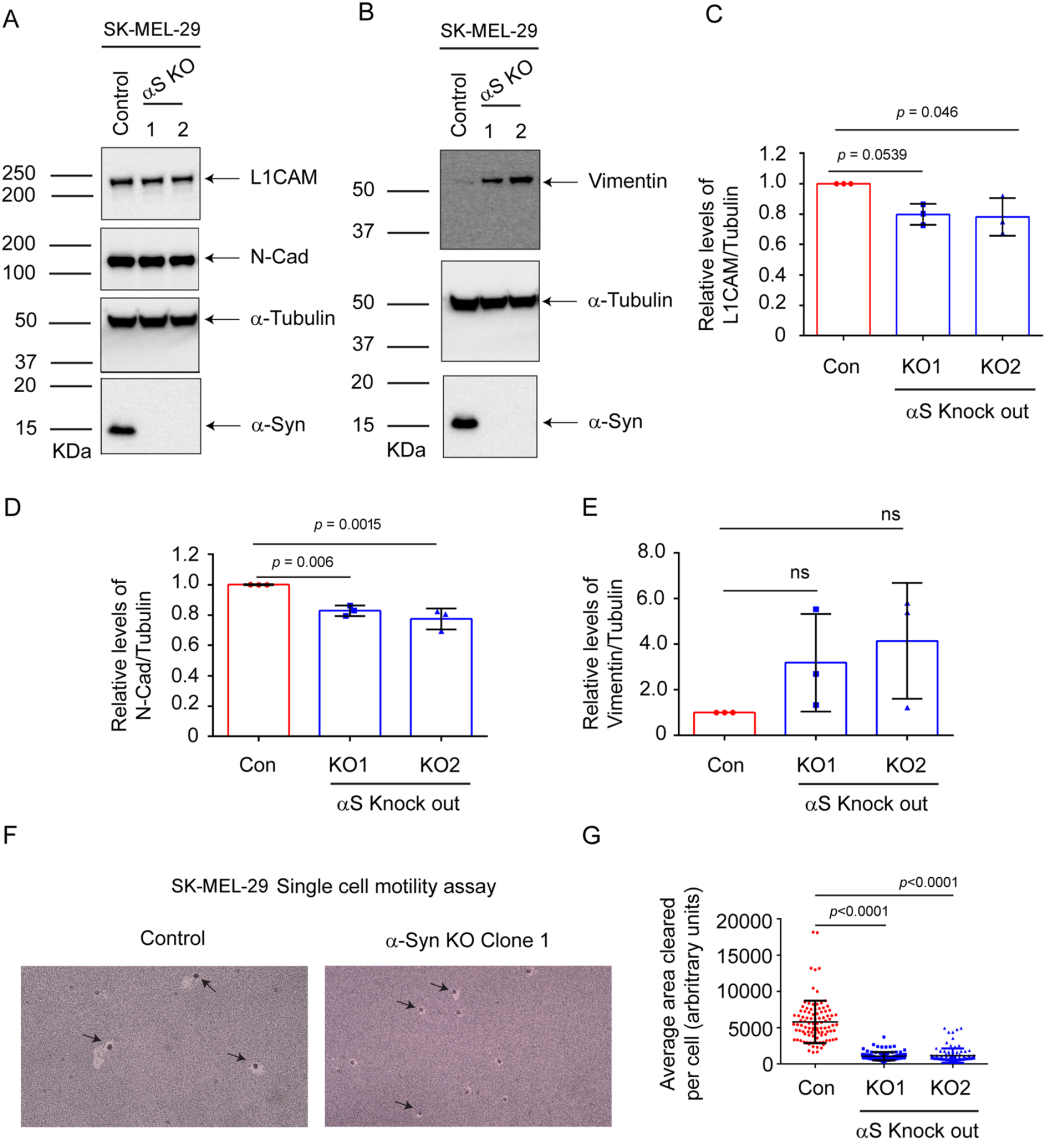
Loss of α-syn decreases L1CAM and N-cadherin and motility in SK-MEL-29 cells. (**A**) Representative Western blots of L1CAM, N-Cad, α-syn, and α-tubulin in lysates of the control and α-syn KO cells cultured in vitro. (**B**) Representative Western blots of vimentin, α-syn, and α-tubulin. Quantitative data showing the relative protein levels of L1CAM (**C**), N-Cad (**D**), and vimentin (**E**) normalized to α-tubulin. All the experiments were repeated with at least with three biological replicates (n = 3). (**F**) Representative brightfield microscope images of phagokinetic tracks created by control and KO α-syn cells on colloidal gold-coated wells were acquired using a light microscope with a ×10 objective. The individual phagokinetic tracks represented by black arrow marks were measured using ImageJ software and represented in (**G**) At least 33 tracks per experimental condition were randomly chosen for quantification. A total of 100 tracks for n = 3 per experimental group were used for statistical analysis. Values in plots (**C–E, G**) are mean ± s.d. *P*-values were determined by a one-way ANOVA, Dunnett post hoc test.

We knocked out *SNCA* in the human cutaneous cell line SK-MEL-29 and used the parental cells and two *SNCA*-KO clones (Table 1) in the following experiments. Western blotting was used to probe the expression of L1CAM, N-cadherin, vimentin, α-syn, and α-tubulin in cell extracts (Fig. 4A, B; Supplementary Fig. S7, uncropped blots). Loss of α-syn expression caused a 20% decrease in the expression of L1CAM in both *SNCA*-KO clones compared to control cells (Fig. 4A, C), a 20% decrease in N-cadherin (Fig. 4A, D), but no change in vimentin (Fig. 4B, E). Although the decreases in L1CAM and N-cadherin expression were modest, nevertheless, three of the four changes were statistically significant. We also conducted the colloidal gold single-cell motility assay on SK-MEL-29 parental cells and two *SNCA*-KO clones. The control cells had much greater motility than either of the two KO clones, and images of the tracks for control and KO1 are shown in Fig. 4 F, and the tracks for KO2 are shown in Supplementary Fig. S7. The plot of the quantified area of single cell tracks showed an 80% decrease in motility (*P* < 0.0001) for each KO clone (Fig. 4G).

### Expressing α-syn in SH-SY5Y increases L1CAM and motility

**Figure 5:**
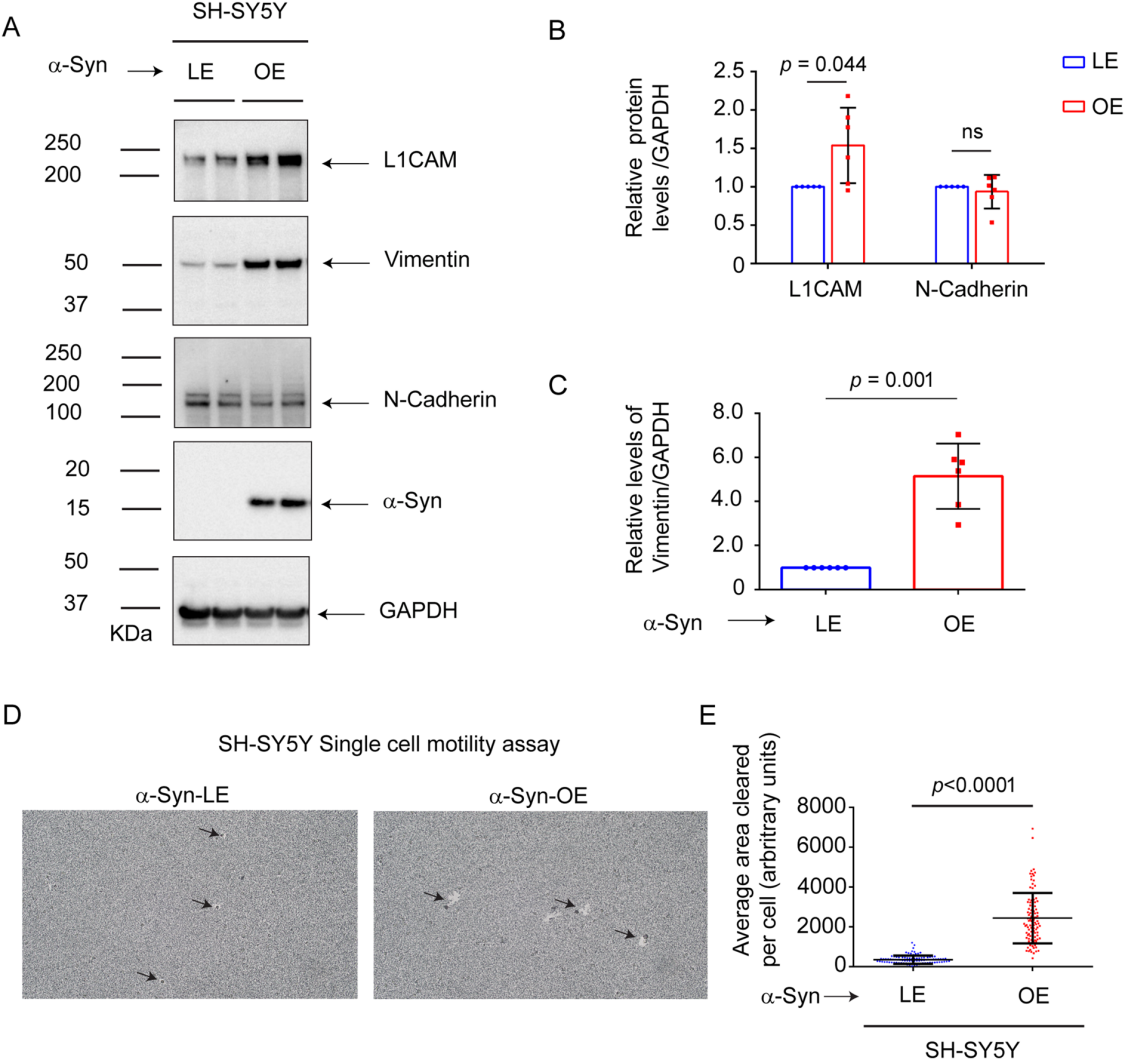
Expressing α-syn in SH-SY5Y cells increases L1CAM and motility. **(A**) Representative western blots of L1CAM, vimentin, N-Cad, α-syn, and α-tubulin in lysates of control and SH/αS cells cultured in vitro. Quantitative analyses representing the relative protein levels were measured by normalizing the band intensities of L1CAM and N-Cad (**B**) and vimentin (**C**) to α-tubulin. All the experiments were repeated with at least with three biological replicates (n = 3–6). (**D**) Representative brightfield microscope images of phagokinetic tracks created by control and α-syn overexpressing SH-SY5Y cells on colloidal gold-coated wells. The images were acquired using a light microscope with a ×10  objective. The individual phagokinetic tracks represented by black arrow marks were measured using ImageJ software and represented in (**E**). At least 33 tracks per experimental condition were randomly chosen for quantification. A total of 100 tracks for n = 3 per experimental group were used for statistical analysis. Values in plots (**B**,**C**,**E**) are mean ± s.d. *P*-values were determined by a two-sided Student’s t test.

Given that the loss of α-syn expression decreases L1CAM, N-cadherin and cell motility in two melanoma cell lines, we asked whether the reverse would be true: Does expressing α-syn in cells that lack α-syn expression increase the expression of pro-oncogenic adhesion proteins and concomitantly increase cell motility? To test this idea, we used the human neuroblastoma cell line SH-SY5Y, which has no detectable α-syn, and SH-SY5Y cells (SH/+αS) that stably express wild-type α-syn (Table 1). L1CAM, N-cadherin, vimentin, α-syn, and GAPDH levels in the cell extracts were probed by Western blotting (Fig. 5A; Supplementary Fig. S8, uncropped blots), yielding the following results. First, α-syn was robustly expressed in the SH/+αS cells but not in the parental line (Fig. 5A, panel 4). Second, the level of L1CAM level was 54% (*P* = 0.044) higher in the SH/+αS cells than in the parental cells (Fig. 5A, panel 1; Fig. 5B). Third, N-cadherin levels were similar in the two cell lines (*P* = 0.508) (Fig. 5A, panel 3; Fig. 5B). Fourth, the level of vimentin was 415% (*P* = 0.001) higher in the SH/+αS cells than in the parental cells (Fig. 5A, panel 2; Fig. 5C).

The single-cell motility of the two SH-SY5Y cell lines was also measured. SH/+αS cells exhibited robust single-cell motility during the 24 h incubation period compared to the parental cells (Fig. 5D). The plot of the quantified area of single cell tracks shows that expressing α-syn in the SH-SY5Y cells significantly increased the single cell motility of such cells compared to control cells. Strikingly, the SH/+αS cells exhibited a 597% (*P* < 0.0001) increase in single-cell motility compared to the parental cells (Fig. 5E).

### Kinetic modeling of effects of α-syn on vesicle trafficking

**Figure 6:**
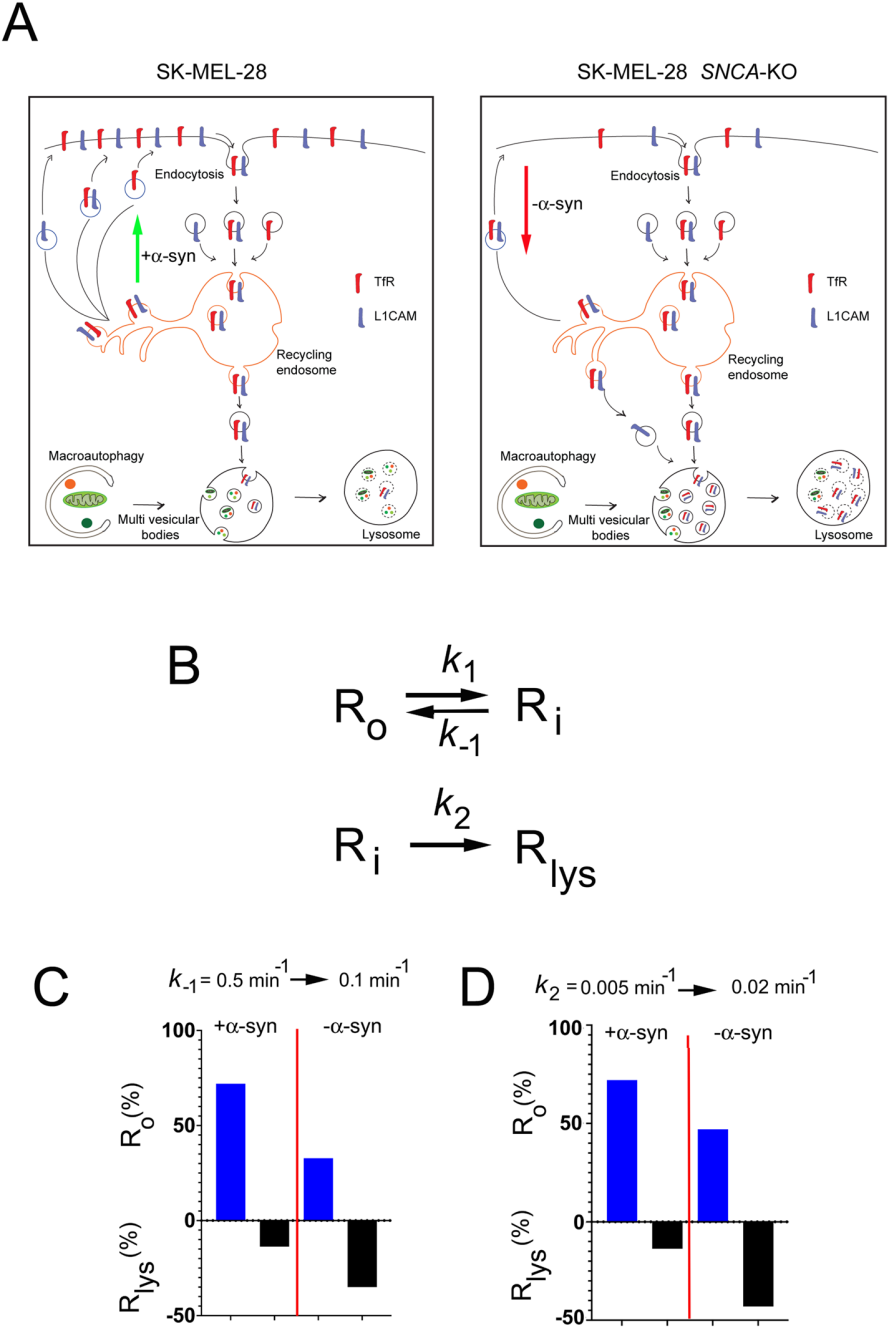
Proposed model and simulations of the effect of α-syn on receptor recycling. (**A**) Left-hand panel: model where α-syn accelerates the trafficking of internalized vesicles containing L1CAM and TfR to the plasma membrane. Right-hand panel: model where loss of α-syn expression results in fewer L1CAM and TfR on the plasma membrane. (**B**) Proposed reactions that model vesicle trafficking. R_o_, R_i_, and R_lys_ is a receptor molecule on the cell surface, in an internalized vesicle, and in the lysosome, respectively. α-syn can differentially affect these three reactions. See simulation results in the Supplementary Simulations file. (**C**) The plot shows the percent of R_o_ and R_lys_ after 180 min of simulation for the following reactions. +α-syn case: k_1_ = 0.1 min^−1^, k_−1_ = 0.5 min^−1^, k_2_ = 0.005 min^−1^. −α-syn case: k_1_ = 0.1 min^−1^, k_−1_ = 0.1 min^−1^, k_2_ = 0.005 min^−1^. (**D**) The plot shows the percent of R_o_ and R_lys_ after 180 min of simulation for the following reactions. + α-syn case: k_1_ = 0.1 min^−1^, k_−1_ = 0.5 min^−1^, k_2_ = 0.005 min^−1^. −α-syn case: k_1_ = 0.1 min^−1^, k_−1_ = 0.5 min^−1^, k_2_ = 0.02 min^−1^.

Classic experiments conducted 40 years ago gave an exquisite account of the kinetics of the internalization of transferrin and the transferrin receptor in a human hepatoma cell line ^47,48^. One of these groups even proposed a model for internalization and recycling that has a default pathway to the lysosome^48^. Their model is an excellent model for the present work (see Fig. 7 of ref ^48^). We now know that TfR and L1CAM each has a cytosolic endocytic recycling sequence ^32,49^, each binds to AP-2, which is a clathrin adapter protein that binds to the recycling sequence, each undergoes clathrin-mediated endocytosis ^32,50^, and they co-localize during clathrin-mediated internalization^32^, as captured in the model in Fig. 6A.

We sought to understand how α-syn affects receptor trafficking by conducting simulations. Based on this previous work on TfR trafficking, the simplest model for the decrease in L1CAM and TfR in *SNCA*-KO cells relative to control cells are the two reactions (also shown in Fig. 6B),

1 $${\text{R}}_{{\text{o}}} \underset{{{\text{k}}_{ - 1} }}{\overset{{{\text{k}}_{1} }}{\rightleftharpoons}}{\text{R}}_{{\text{i}}} \mathop{\longrightarrow}\limits^{{{\text{k}}_{2} }}{\text{R}}_{{{\text{lys}}}}$$

where R_o_, R_i_, and R_lys_ are the plasma membrane-bound receptor, internalized receptor in vesicles, and receptor in the lysosome, respectively. We propose that Eq. (1) can be modeled by small molecules reacting in solution according to

2 $${\text{A}} \underset{{{\text{k}}_{ - 1} }}{\overset{{{\text{k}}_{1} }}{\rightleftharpoons}}{\text{B}}\mathop{\longrightarrow}\limits^{{{\text{k}}_{2} }}{\text{C}}$$

Espenson^51^ solved the differential equations associated with the reactions in Eq. (2), and we used those solutions to probe the role of α-syn in receptor trafficking in two simulations (see “Materials and methods” for details), as follows.

#### Simulation 1

We stipulated that α-syn accelerates the fusion of internalized vesicles with the plasma membrane (R_i_ → R_o_) more than it accelerates endocytosis (R_o_ → R_i_), i.e., k_-1_ > k_1_. This would be an obvious way to increase or maintain the number of receptors on the cell surface. In this model, loss of α-syn expression would therefore decrease the rate that internalized vesicles fuse with the plasma membrane. To simulate this proposed catalytic action of α-syn, one simulation was conducted with k_-1_ = 0.5 min^−1^ and k_1_ = 0.1 min^−1^; whereas the other simulation where α-syn expression is absent was conducted with k_−1_ = k_1_ = 0.1 min^−1^ (in each of these simulations, k_2_ = 0.005 min^−1^). These simulations revealed that *losing α-syn* decreases the number of receptors on the cell surface (72% → 33%) and increases the number in the lysosome (14% → 35%) (Fig. 6C).

#### Simulation 2

We stipulated that α-syn inhibits the transfer of internalized vesicles into the lysosome (R_i_ → R_lys_). It follows that loss of α-syn expression would therefore increase the rate that internalized vesicles transfer into the lysosome. To simulate the proposed inhibitory action of α-syn, one simulation was conducted with k_2_ = 0.005 min^−1^; whereas the other simulation where α-syn expression is absent was conducted with k_2_ = 0.02 min^−1^ (in each of these simulations, k_−1_ = 0.5 min^−1^ and k_1_ = 0.1 min^−1^). These simulations show that *losing α-syn* decreases the number of receptors on the cell surface (72% → 47%) and increases the number of receptors transferred to the lysosome (14% → 44%) (Fig. 6D). Overall, our simulations show that α-syn can alter the number of L1CAM and/or TfR molecules on the cell surface in two very different ways.

## Discussion

The major findings in this report are that (i) loss of α-syn expression in two human cutaneous melanoma cell lines significantly decreases L1CAM, N-cadherin, and cell motility (Figs. 1B, C, E, 4A, C, G)). (ii) Loss of α-syn expression stimulates the degradation of L1CAM in the lysosome (Fig. 3). (iii) An increase in α-syn expression in SH-SY5Y cells increases L1CAM and cell motility (Fig. 5A, B, D, E).

L1CAM is a 200–220 kDa transmembrane glycoprotein that is a member of the immunoglobulin superfamily that has a myriad of activities in the adult nervous system, including neurite outgrowth, migration, adhesion, and neuronal differentiation (for reviews see ^52–54^). L1CAM has been implicated in synaptic plasticity^52^, and, curiously, α-syn has been implicated in synaptic plasticity^40^. L1CAM, which is upregulated in several cancers of neuroectodermal and neural crest origin^55^, including melanomas, is recognized as a tumor antigen involved in motility^41–43^. Ernst et al. found that knocking down L1CAM significantly reduces metastasis in a xenograft model of human melanoma^56^. Herein, we found here that knocking out *SNCA* significantly reduces the level of L1CAM relative to control cells that express α-syn, and the decreased level of this adhesion protein likely contributes to the reduction in cell motility in two melanoma cell lines and one neuroblastoma cell line.

The simulations show that α-syn can increase (or maintain) the amount of L1CAM and or TfR on the cell surface by either promoting the trafficking or fusion of vesicles with the plasma membrane or by inhibiting the fusion of internalized vesicles with the lysosome (Fig. 6). It is theoretically possible that α-syn could increase (or maintain) the amount of L1CAM and TfR on the cell surface by inhibiting endocytosis but given that synuclein has been shown to promote endocytosis^26^ we rejected the idea that synuclein would act by inhibiting endocytosis. Clearly, high resolution microscopy experiments of labeled TfR or labeled L1CAM in cells with and without α-syn expression are needed to decipher the specific reaction or reactions affected by α-syn.

Turriani and colleagues^29^ recently suggested that “there is an inverse molecular link between PD and melanoma and that proteins that are “detrimental players” in PD are “beneficial players” in melanoma because their functions confer significant survival benefits to primary and metastatic melanoma.” Turriani showed that the compound anle128b, which disrupts the prion-like oligomers of α-syn^29^ , protects neurons from α-syn-induced cell death, but in contrast, this compound promotes massive cell death of the WM983-B melanoma cell line. Treating WM983-B cells with anle138b resulted in reduced levels of aggregated α-syn, morphological changes, and disruptions in the mitochondrial membrane potential and autophagy. It was concluded that dissolving aggregated, prion-like forms of α-syn dysregulates autophagy, which suggests that these unusual, aggregated forms of α-syn promote autophagy. Our study and the Turriani study are quite different, in that, Turriani compared melanoma cells that contained aggregated, prion-like forms of α-syn to the same cells where the aggregates were dissolved; whereas we compared melanoma cells that express α-syn to those that do not. On the other hand, each study independently converged on the endolysosomal system, i.e., how synuclein affects lysosome activity and endolysosomal trafficking. More work is needed to decipher the role of α-syn in melanoma.

E- and N-cadherin are Ca^++^-dependent cell–cell adhesion transmembrane glycoproteins^57^. The conserved cytoplasmic tails of these two proteins interact with networks of proteins involved in different cell signaling pathways. The ectodomain of E-cadherin forms homotypic dimers with neighboring cells. N-cadherin is similar in structure to E-cadherin, but its cytoplasmic tail interacts with a different set of proteins^58^. One of the hallmarks of the EMT is the upregulation of N-cadherin followed by the downregulation of E-cadherin^58^. In our hands, knocking out α-syn expression in the two melanoma cell lines significantly decreased the level of N-cadherin but not E-cadherin (Figs. 1A, C, E, 4A, C, D). It is as if loss of α-syn expression partially reverses an ‘EMT-like’ phenomenon.

Renal tubular epithelial cells, conditional knockout mice, and clinical samples of human renal tissue were recently used to assess the role of renal tubular epithelial α-syn in kidney fibrosis^59^. Bozic and colleagues found that treating HK-2 renal cells with transforming growth factor beta 1 (TGF-β1), which mediates fibrosis signaling in renal epithelial cells, changed the epithelial phenotype (loss of cobblestone morphology), decreased E-cadherin, and increased alpha-smooth muscle actin (α-SMA) and vimentin. TGF-β1 concomitantly induced a dose-dependent decrease in *SNCA* mRNA and α-syn expression. Given the co-occurrence of the loss of the epithelial phenotype and the dysregulation of α-syn expression, the authors hypothesized that α-syn has a role in maintaining the epithelial phenotype of renal proximal tubular epithelial cells (RPTECs) in vitro. Their hypothesis was supported by their discovery that overexpressing α-syn in HK-2 cells inhibited TGF-β1-induced increases in α-SMA and vimentin in vitro. They went on to show that α-syn modulates the activation of ERK1/2, Akt and p38 in vitro, that TGF-β1decreases α-syn expression via activation of the MAPK-p38 axis, and that loss of α-syn accelerates profibrotic gene expression. Our group had earlier shown that α-syn inhibits the stress-induced phosphorylation of p38 (and JNK and c-Jun) in SH-SY5Y cells^60^. Their conclusion was that α-syn plays a role in maintaining the epithelial phenotype of RPTECs. Of course, it is hard to compare our results to those of Bozic because we used non-epithelial-derived cancer cells, whereas Bozic used non-dividing renal epithelial cells. On the other hand, these two studies reveal the Dr. Jekyll and Mr. Hyde-like nature of α-syn: α-syn supports an epithelial phenotype of renal cells, whereas it supports a ‘mesenchymal-like’ phenotype of melanoma and neuroblastoma cells.

In sum, we have shown that loss of α-syn expression in two human cutaneous melanoma cell lines results in significant decreases in two adhesion proteins, L1CAM and N-cadherin, and concomitant significant decreases in motility. We propose that α-syn is pro-survival to melanoma (and likely neuroblastoma) because it promotes the efficient vesicular trafficking of L1CAM to the plasma membrane, which in turn promotes invasion, migration, and motility.

## Materials and methods

### Cell lines and cell culture

SK-MEL-28 and SK-MEL-29 cells were purchased from American Type Culture Collection (ATCC, Manassas, VA) and from Sloan-Kettering Memorial Center, respectively, and propagated in DMEM supplemented with 10% fetal bovine serum (FBS) and 1% penicillin–streptomycin. The human neuroblastoma cell line SH-SY5Y over expressing α-syn (SH/αS) and control SH-SY5Y cells were a kind gift of Dr. Joseph R Mazzulli (Northwestern University) and propagated in Opti-MEM supplemented with 10% fetal bovine serum (FBS) and 1% penicillin–streptomycin. CRISPR/Cas9 genome editing was used to target *SNCA* in SK-MEL-29 cells as described previously^31^ for SK-MEL-28 cells using α-syn CRISPR/Cas9 knockout plasmid (Santa Cruz Biotechnology # sc-417273-NIC). Lentivirus particles expressing human α-syn under cytomegalovirus (CMV) promoter (Applied Biological Materials, Inc, Canada) was used to re-express α-syn in SK-MEL-28 KO cells as described previously^31^. For proteasome and autophagy inhibition experiments cells were treated with 10 µM MG132 (Thermofisher Scientific, # M7449) for 6 h and 50 nM bafilomycin A1 (Millipore, # B1793) for 5 h, respectively, in growth medium. The cell lines were authenticated and tested for mycoplasma contamination using MycoAlert® Mycoplasma Detection Kit (# LT07-318).

### Western blotting

Preparation of cell lysates, SDS/PAGE, and western blot analysis was carried out as described previously^31^. Briefly, the cells were lysed in RIPA lysis buffer (50 mM Tris HCl, pH 7.4, 1% NP-40, 0.5% sodium deoxycholate, 0.1% SDS, 5 mM EDTA). The lysates were centrifuged (13,000 rpm/30 min/4 °C), and the protein concentrations of the supernatants were determined using DC™ Protein Assay Kit (Bio-Rad #5 000 112). Samples containing equal concentrations of protein (30 μg) were treated with dithiothreitol (Invitrogen™ NuPAGE™ Sample Reducing Agent # NP0004) and boiled for 10 min at 70C in NuPAGE™ LDS Sample Buffer (#NP000). Proteins were separated by sodium dodecyl sulfate Bis–Tris polyacrylamide gel electrophoresis (SDS-PAGE) (NuPAGE™ 4 to 12%, Bis–Tris precast polyacrylamide gel, Invitrogen # NP0323BOX) and afterward transferred to polyvinylidene difluoride (PVDF) membrane using (Trans-Blot^®^ Turbo™ Mini PVDF Transfer Pack, Bio-Rad # 1704156). After blocking the membranes with 5% blotto (G-Biosciences Blot-Quikblocker # 786-011) in phosphate-buffered saline containing 0.1% (v/v) Tween-20 (PBST) for 1 h at room temperature, the membranes were often cut into strips and the individual strips were hybridized with indicated primary antibodies overnight at 4 °C followed by incubation with respective horse radish peroxidase (HRP) conjugates. The immunoreactive bands were visualized using an enhanced chemiluminescence substrate (Clarity™ Western ECL Substrate, Bio-Rad #170-5060) and the images were acquired using Biorad Chemidoc-MP imaging system. The band intensities of proteins of interest were quantified and represented as relative protein levels normalized to housekeeping protein α-tubulin using ImageJ software. Antibodies (with dilutions) are given in Table 2.

**Table 2:** Antibodies used in this study.

Antibody	Catalog	Company	Application	Dilution
α-synuclein monoclonal (mouse)	610786	BD Biosciences	Western blot	1:1000
LC3-II polyclonal (rabbit)	2775S	Cell Signaling Technology	Western blot	1:2000
Monoclonal (mouse)α-tubulin	T9026	Millipore Sigma	Western blot	1:2000
N-cadherin monoclonal (mouse)	sc-59987	Santa Cruz	Western blot	1:1000
Vimentin monoclonal (mouse)	sc-6260	Santa Cruz	Western blot	1:1000
E-cadherin monoclonal (rabbit)	3195	Cell Signaling Technology	Western blot	1:1000
L1CAM antibody	NB100-2682	Novus Biologicals	Western blot	1:1000
HRP-conjugated anti-rabbit	sc-516102	Santa Cruz	Western blot	1:2000
HRP-conjugated anti-mouse	sc-2357	Santa Cruz	Western blot	1:2000

### Migration and invasion assays

The invasion and metastatic potential of the cell lines in Table 1 was determined by transwell assays using Boyden chambers with an 8-μm pore size (Costar; Corning, Inc. # CL3464). Briefly, the cells were suspended in serum-free DMEM and were seeded onto the apical chamber with (invasion assay) and without (migration assay) matrigel (Corning, Inc. #356234), and 750 μl complete medium containing 10% FBS was added to the lower chamber of the transwell for 24 h. After incubation at 37 °C for 24 h, the cells migrated/ invaded through the lower surface and were fixed with 4% paraformaldehyde for 5 min and then stained with 0.1% crystal violet for 10 min to allow the cells to be visualized. The cells in the upper chamber of the transwell were carefully removed with a cotton swab, and the images of cells in the lower chamber of the transwell were taken using Olympus inverted light microscope.

### Phagokinetic single-cell motility assay

The motility of control and *SNCA*-KO melanoma cell lines was measured by tracking the ability of cells to clear gold from their path^38^. For this, six-well plates were coated with 2 ml of 1% BSA, incubated for 3 h in a humidified CO_2_ incubator at 37 °C, and washed with absolute ethanol. The wells were then coated with a homogenous layer of colloidal gold solution, prepared as follows: A total of 3.85 mL of sterile H_2_O, 630 µL of 14.5 mM AuHCl4, and 2.1 ml of 36.5 mM Na_2_CO_3_, followed by boiling at 100 °C for 5 min and addition of 0.1% of formaldehyde. The colloidal gold-coated plates were incubated in CO_2_ incubator at 37 °C for 24 h, and cells were seeded at a final number 1 × 10^3^ cells per well in a complete medium containing 10% FBS. After 24 h, the wells were imaged using Olympus inverted light microscope, and the tracks were quantified using ImageJ Software.

### RNA extraction, cDNA preparation, and qPCR

Total RNA was extracted from cells using E.Z.N.A column-based total RNA kit (Omega BioTek) following the manufacturer’s instructions. The concentration and quality of the extracted RNA were determined on a NanoDrop spectrophotometer (Thermo Scientific). cDNA was synthesized from total purified RNA (1 μg) from each sample by using iScript cDNA synthesis kit (Bio-Rad) according to the manufacturer’s protocol. qPCR was performed using Applied Biosystems TaqMan™ Gene Expression Assays with primer/probe sets for *SNCA* (Hs00240906), *L1CAM* (Hs01109748), *GAPDH* (Hs02786624). ΔΔCT method was adopted to calculate the relative amount of each mRNA normalized to the housekeeping gene GAPDH. Data were analyzed using the comparative CT method, and the fold change was calculated using the 2^−ΔΔCT^ method using Bio-Rad CFX384 Touch Real-Time PCR System and software (Bio-Rad). The results were expressed either as the relative log_2_ FC (fold change) relative values.

### Kinetic modelling

Espenson^51^ solved the differential equations associated with the reactions in Eq. (2) for the special case [A](t = 0) = [A]_o_ and [B]_o_ = [C]_o_ = 0. The solutions for the three species are

3 $$[\mathrm{A}](\mathrm{t})=\left(\frac{{\text{k}}_{1}{[A]}_{0}}{{\lambda }_{2}-{\lambda }_{3}}\right)\left[\frac{{\lambda }_{2}-{k}_{2}}{{\lambda }_{2}}exp(-{\lambda }_{2}t)-\frac{{\lambda }_{3}-{k}_{2}}{{\lambda }_{3}}exp(-{\lambda }_{3}t)\right]$$

4 $$[\mathrm{B}](\mathrm{t}) = \left(\frac{{\text{k}}_{1}{[A]}_{0}}{{\lambda }_{2}-{\lambda }_{3}}\right)\left[exp(-{\lambda }_{3}t)-exp(-{\lambda }_{2}t)\right]$$

5 $$\left[ {\text{C}} \right]\left( {\text{t}} \right) \, = \, \left[ {\text{A}} \right]\left( {\text{t}} \right) \, - \, \left[ {\text{B}} \right]\left( {\text{t}} \right)$$

where λ_2_ = ½ (p + q) and λ_3_ = ½(p − q) with p = k_1_ + k_−1_ + k_2_ and q = (p^2^ – 4 k_1_k_2_)^1/2^. We used Eqs. (3), (4), and (5) to determine the values of [R_o_](t), [R_i_](t), and [R_lys_](t), respectively.

The simulations were conducted as follows. (i) Eqs. (3), (4), and (5) were input into an EXCEL file (see Supplementary Simulations.xls). (ii) Values for the rate constants k_1_, k_−1_, and k_2_ were selected. (iii) At t = 0, all receptors were on the plasma membrane [R_o_] = 1.0 μM and [R_i_] = [R_lys_] = 0. (iv) Simulations went for 180 min. (v) No protein synthesis occurred during the simulation. (vi) The basis for these simulations was that α-syn, by its ability to bind to membranes, can affect the magnitude of any one of the three rate constants. The values of [R_o_](t = 180 min) and [R_lys_](t = 180 min) are plotted in Fig. 6C, D. [R_lys_](t = 180 min) values are plotted with a negative sign because the receptors are degraded upon entry into the lysosome.

### Statistical analyses

Hypothesis testing methods included a one-way analysis of variance (ANOVA) with a Dunnett post hoc test when comparing multiple groups to control (Figs. 1 E, G, 2, 3C, D, and 4C–E, G), a two-sided Student's t-test when comparing two groups (Fig. 5 B, C, E), and a one-sided Student's t-test when comparing DMSO versus baf-treated samples (Fig. 3B). All data were analyzed using GraphPad Prism (version 6) software. All values were expressed as mean ± standard deviation (s.d.) of at least three independent experiments (biological replicates). *P*-value of < 0.05 was considered significant.

## Supplementary Information

Supplementary Information.

Supplementary Figures.

## Data Availability

All data from this study are contained within the article and its Supplementary Information.

## References

[1] Dean DN, Lee JC (2021). Linking Parkinson's disease and melanoma: Interplay between alpha-synuclein and Pmel17 amyloid formation. Mov. Disord..

[2] Bose A, Petsko GA, Eliezer D (2018). Parkinson's disease and melanoma: Co-occurrence and mechanisms. J. Parkinsons Dis..

[3] Gao X, Simon KC, Han J, Schwarzschild MA, Ascherio A (2009). Family history of melanoma and Parkinson disease risk. Neurology.

[4] Freedman DM, Travis LB, Gridley G, Kuncl RW (2005). Amyotrophic lateral sclerosis mortality in 1.9 million US cancer survivors. Neuroepidemiology.

[5] Bajaj A, Driver JA, Schernhammer ES (2010). Parkinson's disease and cancer risk: A systematic review and meta-analysis. Cancer Causes Control.

[6] Rugbjerg K, Friis S, Lassen CF, Ritz B, Olsen JH (2012). Malignant melanoma, breast cancer and other cancers in patients with Parkinson's disease. Int. J. Cancer.

[7] Schwid SR (2010). Cancer incidence in a trial of an antiapoptotic agent for Parkinson's disease. Mov. Disord..

[8] Goedert M, Spillantini MG, Del Tredici K, Braak H (2013). 100 years of Lewy pathology. Nat. Rev. Neurol..

[9] Kalia LV, Lang AE (2015). Parkinson's disease. Lancet.

[10] Alegre-Abarrategui J (2019). Selective vulnerability in a-synucleinopathies. Acta Neuropathol..

[11] Polymeropoulos MH (1997). Mutation in the alpha-synuclein gene identified in families with Parkinson's disease. Science.

[12] Spillantini MG (1997). Alpha-synuclein in Lewy bodies. Nature.

[13] Kruger R (1998). Ala30Pro mutation in the gene encoding alpha-synuclein in Parkinson's disease. Nat. Genet..

[14] Martinez-Navarrete GC, Martin-Nieto J, Esteve-Rudd J, Angulo A, Cuenca N (2007). alpha-synuclein gene expression profile in the retina of vertebrates. Mol. Vis..

[15] Nakai M (2007). Expression of alpha-synuclein, a presynaptic protein implicated in Parkinson's disease, in erythropoietic lineage. Biochem. Biophys. Res. Commun..

[16] Abd-Elhadi S, Basora M, Vilas D (2016). Total a-synuclein levels in human blood cells, CSF, and saliva deetrmined by a lipid-ELISA. Anal. Bioanal. Chem..

[17] Matsuo Y, Kamitani T (2010). Parkinson's disease-related protein, alpha-synuclein, in malignant melanoma. PLoS ONE.

[18] Surguchov A (2023). alpha-synuclein and mechanisms of epigenetic regulation. Brain Sci..

[19] Logan T, Bendor J, Toupin C, Thorn K, Edwards RH (2017). alpha-Synuclein promotes dilation of the exocytotic fusion pore. Nat. Neurosci..

[20] Kubo S (2005). A combinatorial code for the interaction of alpha-synuclein with membranes. J. Biol. Chem..

[21] Chandra S, Chen XC, Rizo J, Jahn R, Sudhof TC (2003). A broken alpha-helix in folded alpha-synuclein. J. Biol. Chem..

[22] Mao XB (2016). Pathological alpha-synuclein transmission initiated by binding lymphocyte-activation gene 3. Science.

[23] Shrivastava AN (2015). alpha-synuclein assemblies sequester neuronal alpha 3-Na^+^/K^+^-ATPase and impair Na^+^ gradient. EMBO J..

[24] Sousa VL (2009). alpha-synuclein and its a30p mutant affect actin cytoskeletal structure and dynamics. Mol. Biol. Cell.

[25] Schaser AJ (2019). Alpha-synuclein is a DNA binding protein that modulates DNA repair with implications for Lewy body disorders. Sci. Rep..

[26] Ben Gedalya T (2009). alpha-synuclein and polyunsaturated fatty acids promote clathrin-mediated endocytosis and synaptic vesicle recycling. Traffic.

[27] Weinreb PH, Zhen W, Poon AW, Conway KA, Lansbury PTJ (1996). NACP, a protein implicated in Alzheimer's disease and learning, is natively unfolded. Biochemistry.

[28] Luk KC (2012). Pathological alpha-synuclein transmission initiates Parkinson-like neurodegeneration in nontransgenic mice. Science.

[29] Turriani E (2017). Treatment with diphenyl-pyrazole compound anle138b/c reveals that alpha-synuclein protects melanoma cells from autophagic cell death. Proc. Natl. Acad. Sci. USA.

[30] Baksi S, Tripathi AK, Singh N (2016). Alpha-synuclein modulates retinal iron homeostasis by facilitating the uptake of transferrin-bound iron: Implications for visual manifestations of Parkinson's disease. Free Radic. Biol. Med..

[31] Shekoohi S (2021). Knocking out alpha-synuclein in melanoma cells dysregulates cellular iron metabolism and suppresses tumor growth. Sci. Rep..

[32] Kamiguchi H (1998). The neural adhesion molecule L1 interacts with the AP-2 adaptor and is endocytosed via the clathrin-mediated pathway. J. Neurosci..

[33] Nieto MA, Huang RY-J, Jackson RA, Thiery JP (2016). EMT: 2016. Cell.

[34] Lambert AW, Pattabiraman DR, Weinberg RA (2017). Emerging biological principles of metastasis. Cell.

[35] Davies H (2002). Mutations of the BRAF gene in human cancer. Nature.

[36] Morrison DK (2012). MAP kinase pathways. Cold Spring Harb. Perspect. Biol..

[37] Xicoy H, Wieringa B, Martens GJM (2017). The SH-SY5Y cell line in Parkinson's disease research: A systematic review. Mol. Neurodegener..

[38] Nogalski MT, Chan GCT, Stevenson EV, Collins-McMillen DK, Yurochko AD (2012). A Quantitative evaluation of cell migration by the phagokinetic track motility assay. J. Vis. Exp..

[39] Kim HY (2017). Discovery of potential biomarkers in human melanoma cells with different metastatic potential by metabolic and lipidomic profiling. Sci. Rep..

[40] George JM, Jin H, Woods WS, Clayton DF (1995). Characterization of a novel protein regulated during the critical period for song learning in the zebra finch. Neuron.

[41] Anderson HJ, Galileo DS (2016). Small-molecule inhibitors of FGFR, integrins and FAK selectively decrease L1CAM-stimulated glioblastoma cell motility and proliferation. Cell. Oncol. (Dordr).

[42] Hoja-Lukowicz D (2013). L1CAM from human melanoma carries a novel type of N-glycan with Gal beta 1–4 Gal beta 1-motif. Involvement of N-linked glycans in migratory and invasive behaviour of melanoma cells. Glycoconj. J..

[43] Yang MH (2009). Stimulation of glioma cell motility by expression, proteolysis, and release of the L1 neural cell recognition molecule. Cancer Cell Int..

[44] Cuervo AM, Stefanis L, Fredenburg R, Lansbury PT, Sulzer D (2004). Impaired degradation of mutant alpha-synuclein by chaperone-mediated autophagy. Science.

[45] Zhao L, Zhao J, Zhong K, Tong A, Jia D (2022). Targeted protein degradation: Mechanisms, strategies and application. Signal Transd. Targeted Ther..

[46] Klionsky DJ (2016). Guidelines for the use and interpretation of assays for monitoring autophagy (3rd edition). Autophagy.

[47] Ciechanover A, Schwartz AL, Dautry-Varsat A, Lodish HF (1983). Kinetics of internalization and recycling of transferrin and the transferrin receptor in a human hepatoma cell line. J. Biol. Chem..

[48] Dautry-Varsat A, Ciechanover A, Lodish HF (1983). pH and the recycling of transferrin during receptor-mediated endocytosis. Proc. Natl. Acad. Sci. USA.

[49] Ohno H (1995). Interaction of tyrosine-based sorting signals with clathrin-associated proteins. Science.

[50] Klausner RD (1983). Receptor-mediated endocytosis of transferrin in K562 cells. J. Biol. Chem..

[51] Espenson, J. H. *Chemical Kinetics and Reaction Mechanisms*. 71–72 (McGraw-Hill, 1981).

[52] Duncan BW, Murphy KE, Maness PF (2021). Molecular mechanisms of L1 and NCAM adhesion molecules in synaptic pruning, plasticity, and stabilization. Front. Cell Dev. Biol..

[53] Samatov TR, Wicklein D, Tonevitsky AG (2016). L1CAM: Cell adhesion and more. Prog. Histochem. Cytochem..

[54] Maness PF, Schachner M (2007). Neural recognition molecules of the immunoglobulin superfamily: Signaling transducers of axon guidance and neuronal migration. Nat. Neurosci..

[55] Rawnaq T (2012). L1 is highly expressed in tumors of the nervous system: A study of over 8000 human tissues. J. Surg. Res..

[56] Ernst AK (2018). Knockdown of L1CAM significantly reduces metastasis in a xenograft model of human melanoma: L1CAM is a potential target for anti-melanoma therapy. PLoS ONE.

[57] Shapiro L (1995). Structural basis for homophilic adhesion by cadherins. Nature.

[58] Loh CY (2019). The E-cadherin and N-Cadherin switch in epithelial-to-mesenchymal transition: Signaling, therapeutic implications, and challenges. Cells.

[59] Bozic M (2020). Protective role of renal proximal tubular alpha-synuclein in the pathogenesis of kidney fibrosis. Nat. Commun..

[60] Wang S (2012). alpha-Synuclein disrupts stress signaling by inhibiting polo-like kinase Cdc5/Plk2. Proc. Natl. Acad. Sci. USA.

[61] Cuddy LK (2019). Stress-induced cellular clearance is mediated by the SNARE protein ykt6 and disrupted by alpha-synuclein. Neuron.

