# Supplementary material for: Knocking out alpha-synuclein in melanoma cells downregulates L1CAM and decreases motility
Source: Sci Rep. 2023 Jun 7;13:9243. doi: 10.1038/s41598-023-36451-3 (PMC10247798; doi:10.1038/s41598-023-36451-3)
Supplement: Supplementary file 2 — Supplementary Figures. [file 41598_2023_36451_MOESM2_ESM.pdf]

Supplementary Information

Knocking out alpha-synuclein in melanoma cells downregulates L1CAM and decreases motility

Nithya Gajendran[1,#], Santhanasabapathy Rajasekaran[1,#], and Stephan N. Witt[1,2*]

[1]Department of Biochemistry and Molecular Biology, Louisiana State University Health Sciences Center, Shreveport, USA. [2]Feist-Weiller Cancer Center, Louisiana State University Health Shreveport, Shreveport, USA.

#, equal contributions

*To whom correspondence should be addressed: Tel: 318-675-7826; Fax: 318-675-5180; E-mail: stephan.witt@lsuhs.edu

Keywords: alpha-synuclein, epithelial-to-mesenchymal transition, L1CAM, migration, motility, N-cadherin

Supplementary Figure S1

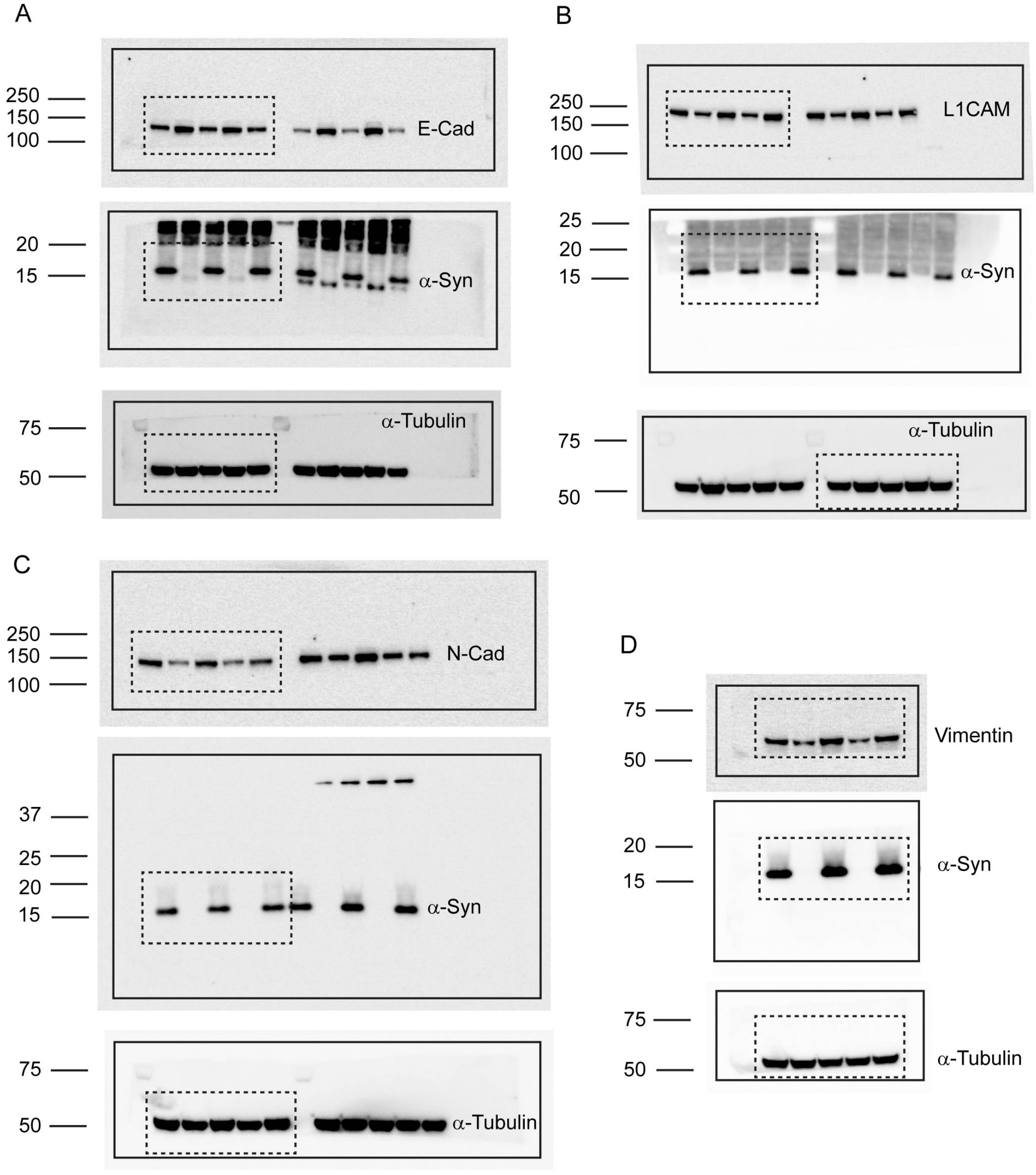

Supplementary Fig. S1

(**A-D**) Full-length original uncontrasted Western blots are shown cropped in Figs. 1 A, B, C, and D. The edges of the blots are represented by a solid rectangle, and the cropped area is represented by a broken rectangle.

Supplementary Figure S2

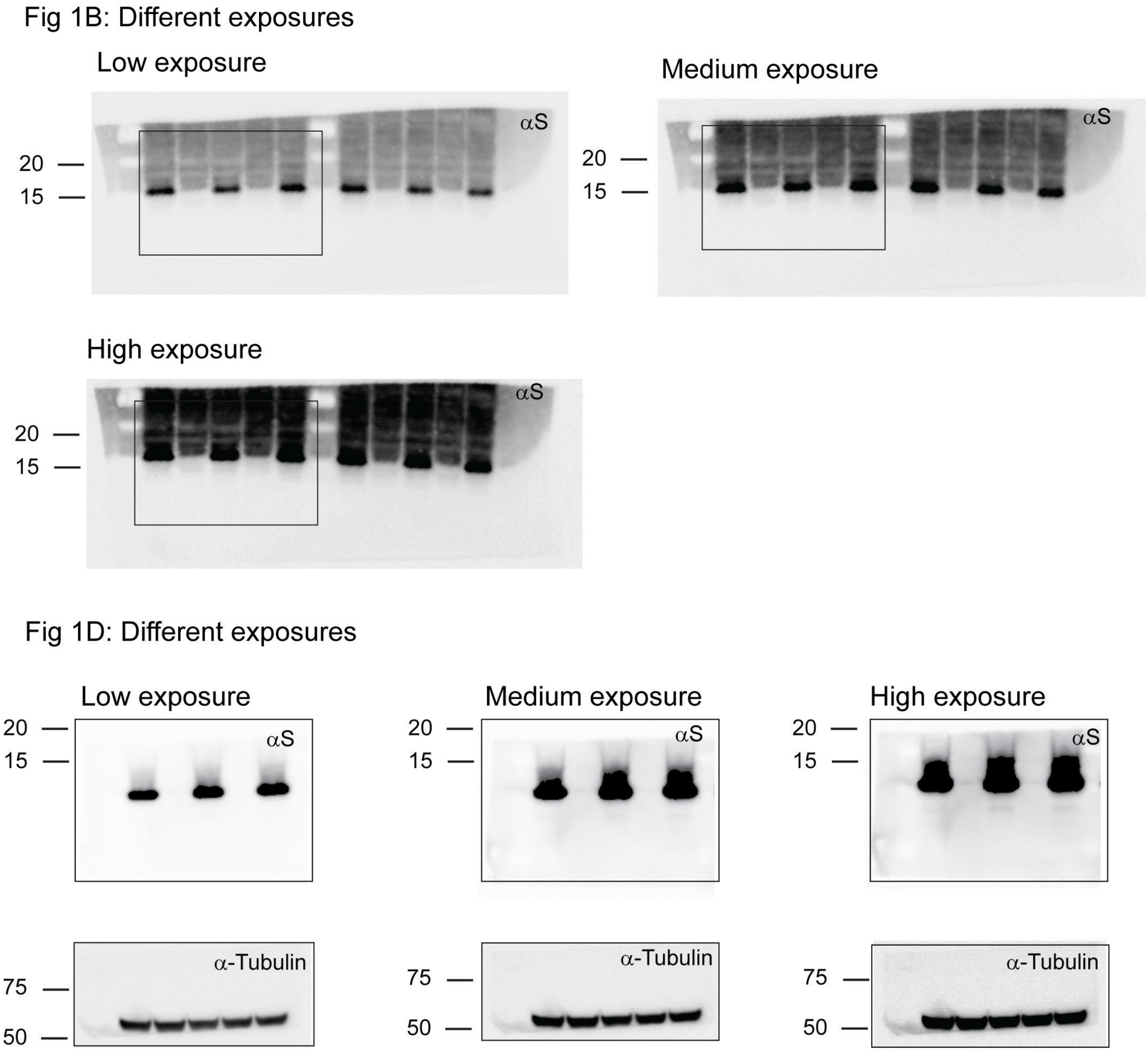

Supplementary Fig. S2

This figure shows several exposures (low, medium, and high) for the Western blots in Fig. 1 B and D.

Supplementary Figure S3

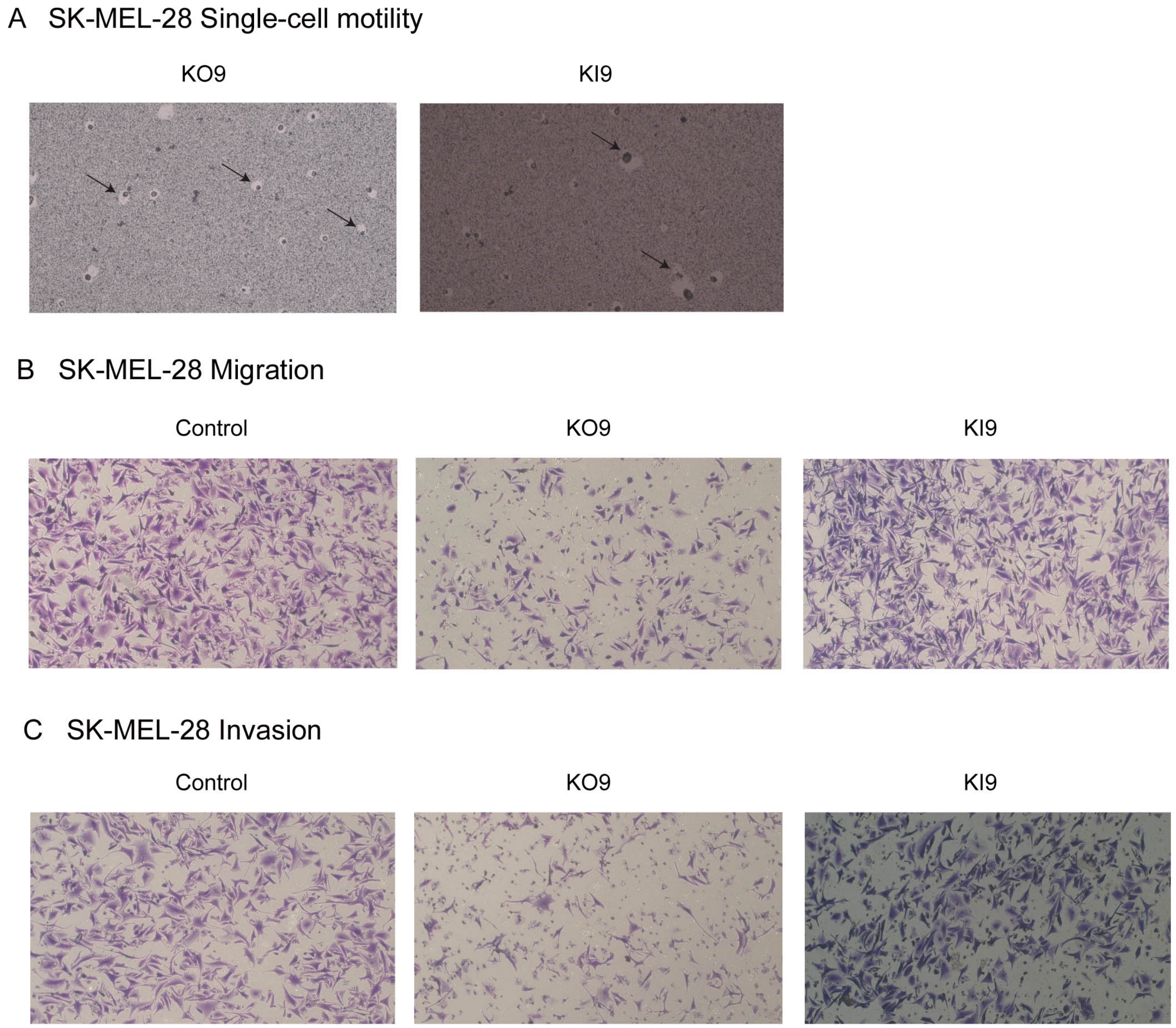

Supplementary Fig. S3

(**A**) Representative light microscope images of phagokinetic tracks created by KO9 and KI9 cells on colloidal gold-coated wells for the data shown in Fig. 1E and F. Black arrows mark individual phagokinetic tracks in respective cell lines. (**B**) Representative images (10X objective) of migrated control, KO9, and KI9 cells (after 24 hours) for the data shown in Fig. 2A. (**C**) Representative images (10X objective) of invaded control, KO9 and KI9 cells (after 24 hours) per 10X field for the data shown in Fig. 2B.

Supplementary Figure S4

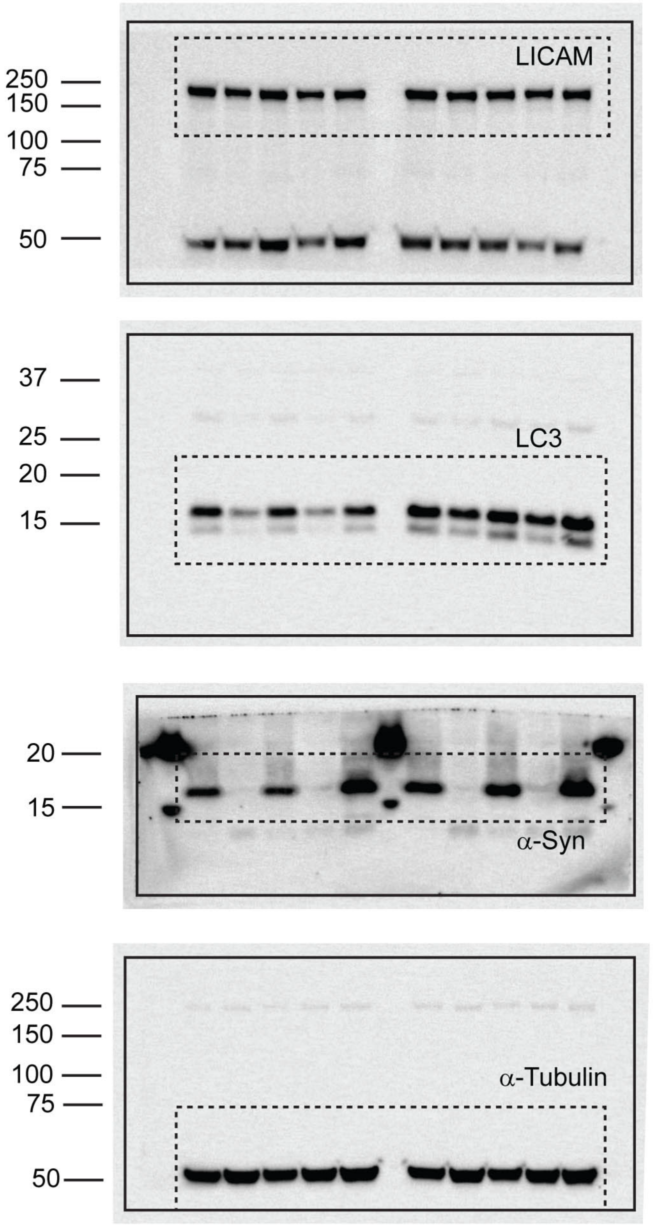

Supplementary Fig. S4.

Full-length, original uncontrasted Western blots are shown cropped in Figs. 3 A. A solid rectangle represented the edges of the blots and the cropped area was represented by a broken rectangle.

Supplementary Figure S5

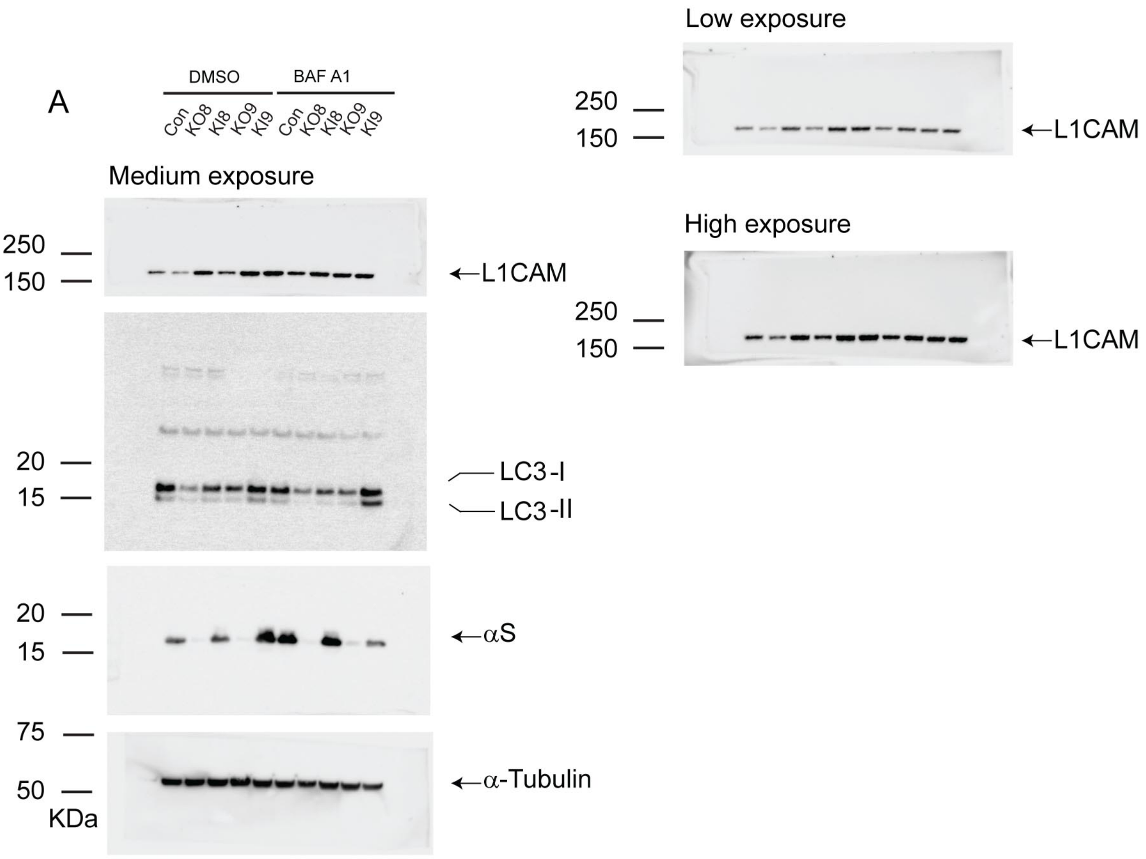

Supplementary Fig. S5

Fig. 3 shows the loss of α-syn promotes the lysosomal degradation of L1CAM. Panel (**A**) in this Supplementary figure shows a Western blot analysis of another biological replicate. Band intensities of this blot were used to construct the plots in Figure 3B and C. Overall, this experiment had 3 biological replicates. Also shown are low and high exposures for the L1CAM blots.

Supplementary Figure S6

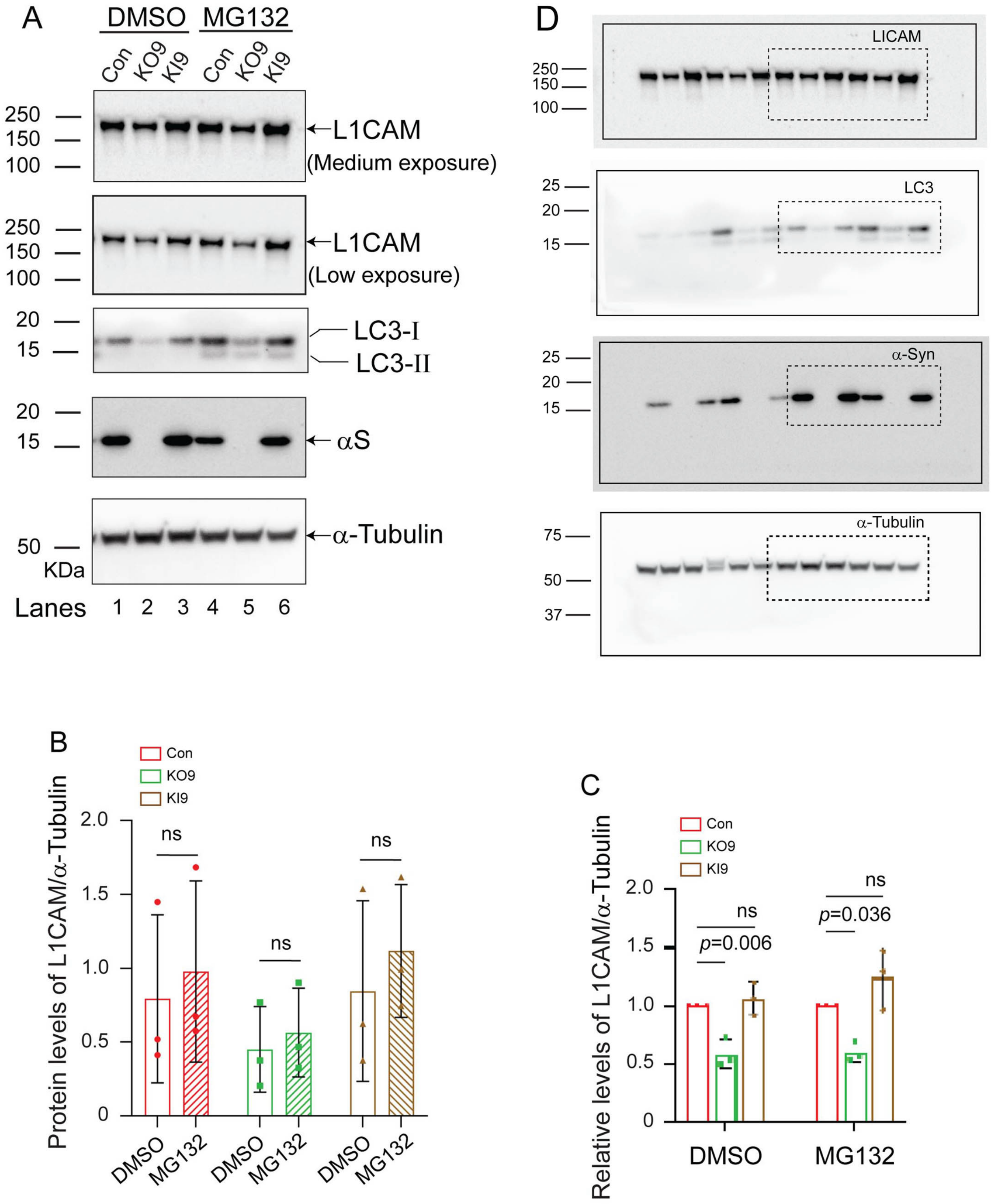

Supplementary Fig. S6

The proteasome does not degrade L1CAM. (**A**) Representative Western blots showing the effect of MG132 (10 μM) on the levels of L1CAM, LC3-I, -II, α-syn, α-tubulin in lysates of the control, KO and KI cells cultured *in vitro*. Band intensities were quantified by densitometry. This experiment was conducted on n=3 biological replicates. (**B**) Plot of L1CAM level in lysates of untreated (DMSO) versus treated (MG132) cells. In this plot, L1CAM (L1) was normalized to α-tubulin (tub), according to $\left({}^{I_{L1}}/_{I_{tub}}\right)$, where I_{L1} and I_{tub} are the average intensities of the respective bands. *P*-values were determined by a one-sided Student's t test. (**C**) Plot of L1CAM level compared by treatment group. In this plot, L1CAM was normalized to α-tubulin, according to $\left({}^{I_{L1}}/_{I_{tub}}\right)_{sample}\left({}^{I_{tub}}/_{I_{L1}}\right)_{control}$. *P*-values determined by a one-way ANOVA with Dunnett post hoc test. (**B**, **C**) Values in plots are mean ± s.d. ns, not significant. (**D**) Full-length, original uncontrasted Western blots which are shown cropped in Fig. S6A. A solid rectangle represented the edges of the blots and the cropped area was represented by a broken rectangle.

Supplementary Figure S7

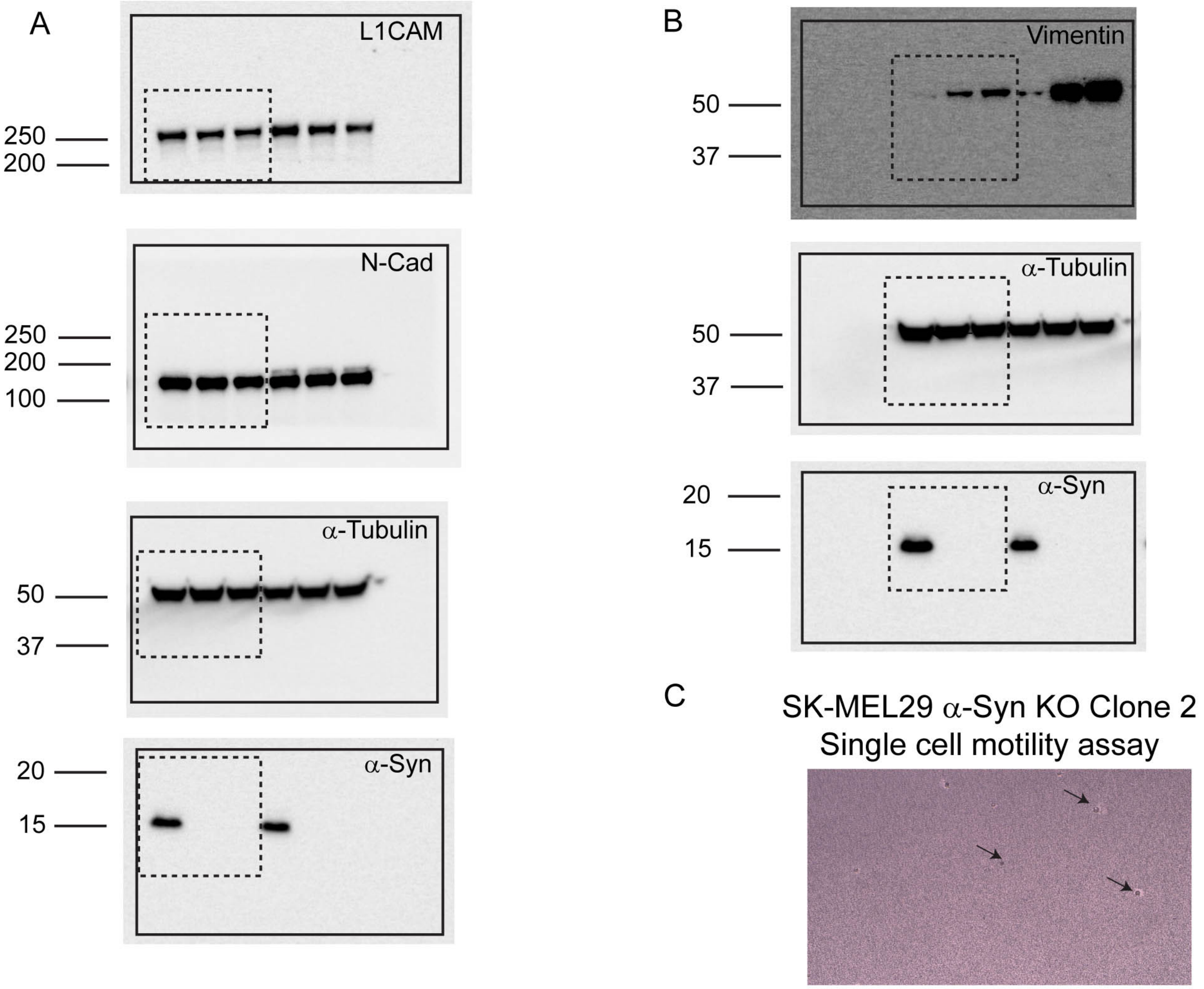

Supplementary Fig. S7

(**A**, **B**) Full-length, original uncontrasted Western blots which are shown cropped in Figs. 4 A and B. The edges of the blots are represented by a solid rectangle and the cropped area is represented by a broken rectangle. (**C**) Representative light microscope images of phagokinetic tracks created by SK-MEL29 α-syn KO Clone 2 on colloidal gold-coated wells for the data shown in Figs. 4F and G. Black arrows mark individual phagokinetic tracks in respective cell lines.

Supplementary Figure S8

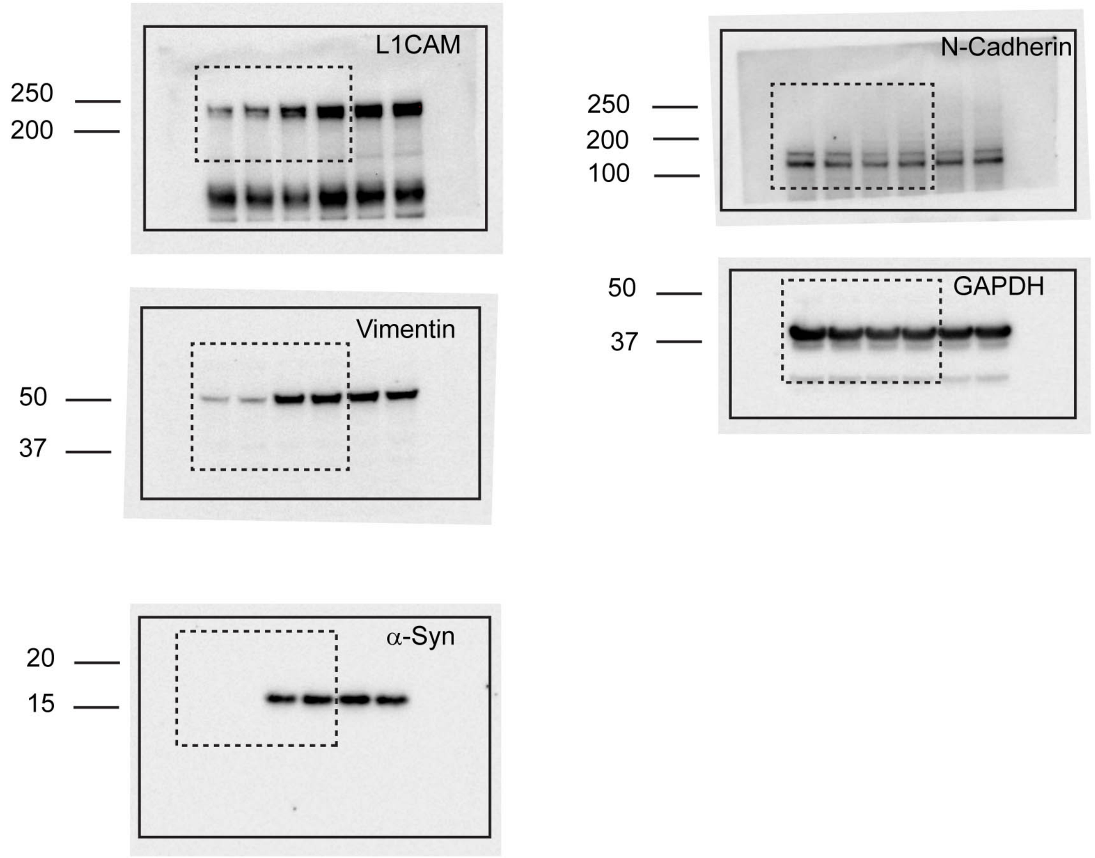

Supplementary Fig. S8

Full-length, original uncontrasted Western blots are shown cropped in Fig. 5A. The edges of the blots are represented by a solid rectangle and the cropped area is represented by a broken rectangle.
